# Scaling beyond the vagus nerve: historical and contemporary progress on electrode-based small-diameter peripheral nerve interfaces

**DOI:** 10.1186/s42234-025-00199-0

**Published:** 2026-02-28

**Authors:** Max Li, Christine Ly, Jinghua Wen, Paritosh Rustogi, Ellis Meng

**Affiliations:** 1 Alfred E. Mann Department of Biomedical Engineering, University of Southern California, Los Angeles, USA; 2 Ming Hsieh Department of Electrical and Computer Engineering, University of Southern California, Los Angeles, USA

**Keywords:** Peripheral nerve interface, Cuff electrode, Intraneural interface, Intrafascicular interface, Regenerative interface

## Abstract

Peripheral nerve interfaces play a central role in bioelectronic medicine. Since the early foundational experiments of Luigi Galvani in the 1770s, there have been over 250 years of development in electrical neuromodulation. Even so, current clinical approaches to interface with peripheral nerves are limited. Bioelectronic interfaces for small, branched nerves are of increasing interest to unlock new therapies and minimize off-target effects. This is facilitated by our growing understanding of peripheral nervous system physiology and advances in new materials and technologies. Therefore, this review examines historical and recent developments in FDA-approved peripheral nerve interfaces and investigational interfaces with an emphasis on approaches to target smaller nerves. Unmet needs in small nerve peripheral nerve interfaces are highlighted, followed by an examination of new strategies being pursued to address them. To conclude, ongoing challenges are summarized, revealing opportunities and prospects for future advancements.

## Introduction

**Fig. 1:**
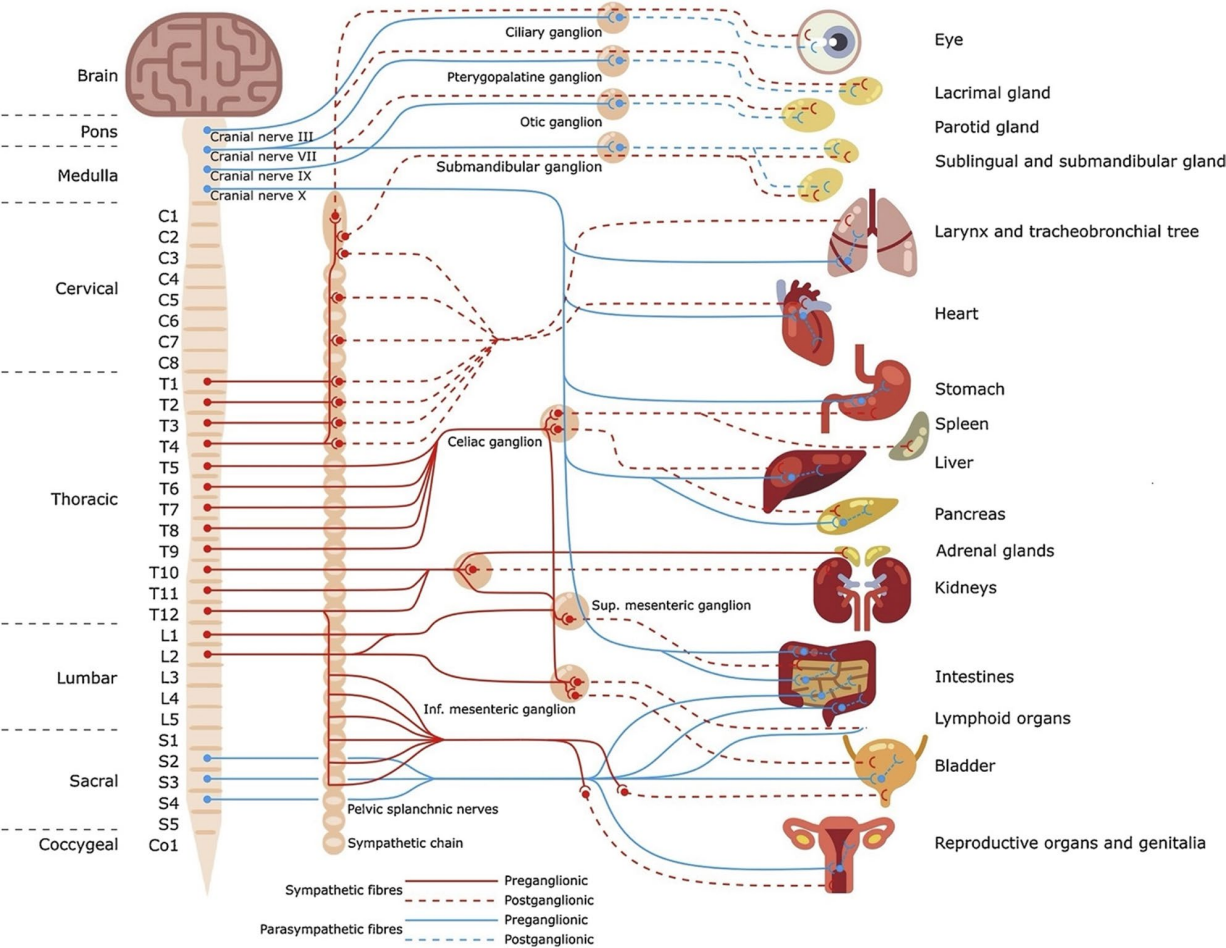
A graphical overview of the human autonomic system, including the innervated organs. Adapted from (Thompson et al. 2019) under CC BY 4.0.

The peripheral nervous system (PNS) consists of a vast network of efferent and afferent nerve fibers, interconnecting the central nervous system with the rest of the body, including organs, muscles, and sensory receptors (Fig. 1) (Hubbard 1974). This peripheral nerve network provides an alternative avenue for the delivery of electrical stimulation to specific targets and the potential to minimize side effects compared to systemic therapies. In the last decade, renewed attention on the physiology of peripheral nerves and the development of application-specific nerve interfaces has been spurred by substantial investment from research agencies and private capital seeking to realize the potential of bioelectronic medicine for a broad range of chronic conditions and diseases.

As of 2024, over 130,000 patients have vagus nerve stimulation (VNS) device implants, making VNS one of the most widely adopted neuromodulation therapies in clinical practice (Zanello et al. 2025). The human cervical vagus nerve, however, contains over 100,000 fibers, with efferent fibers innervating most visceral organs and afferent fibers returning signals to the brainstem (Neuhuber and Berthoud 2021; Paggi et al. 2024; Upadhye et al. 2022). Therefore, stimulation of the entirety of the vagus nerve using an extraneural electrode can result in off-target effects through the indiscriminate activation of nerve fibers and current spread beyond the intended target. Within the same nerve, activation of fibers is size-dependent where larger motor fibers have lower activation thresholds than smaller fibers (McAllen et al. 2018). Consequently, VNS can result in unintended motor or sensory activation including muscle contractions, cough, dysesthesia, intestinal inflammation, heart rate modulation, breathing rate modulation, and other autonomic disturbances (Ben-Menachem 2001; Bielefeldt 2016; Nicolai et al. 2020; Payne et al. 2019).

To avoid such side effects, new therapies targeting branched nerves have been proposed, such as stimulation of the celiac branch of the vagus nerve for hypoglycemia control (Waataja et al. 2022) or S3 and S4 sacral root stimulation for inflammatory bowel disease management (Chen et al. 2024; Liu et al. 2015). Targeting branched peripheral nerves can alleviate chronic pain (Gilmore et al. 2020; Kaye et al. 2021), reduce attention deficit hyperactivity disorder (ADHD) symptoms for young patients (Rubia et al. 2021), and allow paralysis patients and amputees to control prosthetic arms or regain sensation (Navarro et al. 2005). At the same time, new therapies may arise from ongoing studies of different branches, unveiling their arrangement and correlation to organ function (Jayaprakash et al. 2023; Upadhye et al. 2022; Yount et al. 2023). However, translation of promising new therapies involving such nerves measuring 1mm or less in diameter has been impeded by the lack of suitably sized nerve-specific clinical grade interfaces.

**Fig. 2:**
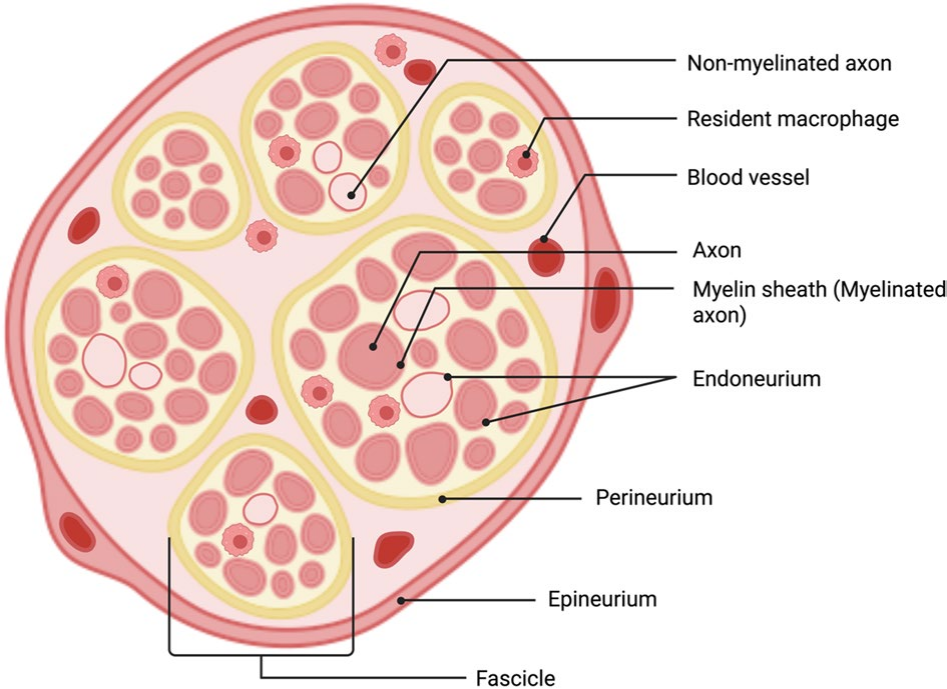
A cross-sectional diagram illustrating the anatomical organization of the peripheral nerve. Peripheral nerves consist of many nerve fiber bundles forming fascicles, each protected by a perineurium sheath, and which are commingled with blood vessels, Schwann cells, and immune cells then wrapped into a cable-like structure by the epineurium. created in BioRender. (Li 2026) https://BioRender.com/ax8r1mx

Prior to a deeper examination of the current state-of-the-art, we briefly revisit peripheral nerve anatomy, the different fiber types, and their function as these factors can influence peripheral nerve interface (PNI) design and the ability to select a target of interest. Peripheral nerves consist of many nerve fiber bundles that form fascicles, each protected by a perineurium sheath (Fig. 2).

These are commingled with blood vessels, Schwann cells, and immune cells then wrapped into a cable-like structure by the epineurium. Fibers may be myelinated or unmyelinated and vary in diameter, both of which affect conduction velocity and activation threshold (Table 1). Although it is easier to activate the larger fiber types, peripheral nerves contain significantly more small-diameter, unmyelinated C-type nerve fibers, generally located in the nerve interior. C-type nerve fibers act as high-intensity nociceptors and afferents (Grinberg et al. 2008) and have been shown to play a significant role in regulating physiological functions such as glycemic control (Waataja et al. 2022), pain transduction (Rydevik et al. 1990) and interoception (Björnsdotter et al. 2010). Despite clinical interest in C-type fibers, activating and recording from them is more challenging due to their higher stimulation thresholds and location within the nerve (Waxman 1980). In addition, larger fibers generate higher amplitude action potentials which are easier to record.

**Table 1:** A summary of the properties and functions of nerve fiber types. Reproduced with permission from (Menorca et al. 2013). Copyright 2013 Elsevier.

Fiber Type	Myelination	Diameter (mm)	Conduction Velocity (m/s)	Location	Function
Aα	Yes	6–22	30–120	Efferent to muscles	Motor
Aβ	Yes	6–22	30–120	Afferent from skin and joints	Tactile, proprioception
Aγ	Yes	3–8	15–35	Efferent to muscle spindles	Muscle tone
Aδ	Yes	1–4	5–30	Afferent sensory nerves	Pain, cold, temperature, touch
B	Yes	1–3	3–15	Preganglionic sympathetic	Various autonomic functions
sC	No	0.3–1.3	0.7–1.3	Postganglionic sympathetic	Various autonomic functions
dC	No	0.4–1.2	0.1–2.0.1.0	Afferent sensory nerves	Various autonomic functions, pain, warm, temperature, touch

The purpose of this review is to examine the historical underpinnings of modern-day clinical devices having electrode-based peripheral nerve interfaces (PNIs) for delivering bioelectronic medicine and then address progress towards realizing smaller nerve interfaces needed for next generation therapies. Design criteria and considerations for interfacing with nerves, available construction materials and fabrication methodologies, and different approaches for small nerve interfacing are reviewed in depth. Finally, additional challenges are discussed, highlighting avenues for future work to advance the development of small PNIs. For a more detailed analysis of the fundamental and practical principles guiding general PNI design, regardless of nerve size, the reader is referred to this review (Larson and Meng 2019).

## A brief historical review of peripheral nerve interface development

The use of electricity as a means of providing therapeutic benefit has been documented since the ancient Egyptians, Greeks, and Romans who used various species of fish as sources of electrical shocks for headaches and pain relief (Heidland et al. 2013; Kane and Taub 1975; Sironi 2011). Advances in the ability to generate and store electricity led to the Leyden jar which played a critical role in an early serendipitous observation by Luigi Galvani; a metal knife served as the neural interface to a frog nerve, conducting an incidental spark during dissection and producing an observable contraction (Kazamel and Warren 2017).

Following Galvani’s work, interest and progress in bioelectricity waxed and waned. Steady progress in devices to manipulate and monitor electricity spurred fundamental advances in electrophysiology but also questionable exploits in electrotherapy. Early work exploring transcutaneous vagal and cervical sympathetic nerve stimulation to treat epilepsy in the 1880s was controversial, producing several adverse side effects including bradycardia and syncope (Lanska 2002). The lack of scientific credibility associated with such early work stifled further development until the latter half of the 20th century when experiments revealed new insights on the neurophysiological mechanisms underlying pain and other conditions.

New clinical applications emerged in the 1960s, including electrical stimulation of the phrenic nerve for diaphragmatic pacing (Judson 1968), pudendal nerve for urinary incontinence, and trigeminal nerve for facial pain (Shelden et al. 1967). Clinical devices leveraged advances in electronics, allowing miniaturization of circuits and fully implantable stimulators. However, the associated early nerve interfaces were decidedly less sophisticated, consisting of simple wires and foils with polymer insulation, and variants of these technologies formed the basis for modern clinical implants. The Food and Drug Administration (FDA) has since granted approval for phrenic nerve stimulator (1987) for upper motor neuron respiratory muscle paralysis (“Diaphragm Pacing System Information,” n.d.), and a vagus nerve stimulator having a multi-turn helical extraneural electrode in the form of a cuff for treating drug-resistant epilepsy in 1997 and treatment-resistant depression in 2005 (Krahl 2012). Sacral nerve stimulators with a quadripolar lead for treating urinary urge incontinence were also approved in 1997 (Spilotros et al. 2024). These represent significant milestones that provided momentum for many subsequent approvals for peripheral nerve stimulators discussed in the next section.

By 1970, rapid advances in semiconductor manufacturing led to the introduction of silicon as a material for planar neural interfaces, initially investigated for use in the brain (Wise et al. 1970). This introduced a radically different method to construct electrode-based interfaces and enabled both precision in manufacturing as well as access to miniature, cellular-scale electrodes. Some of these technologies have been employed in peripheral nerves, although no microfabricated PNIs have made it to FDA approved devices.

## Design of modern clinical devices

**Fig. 3:**
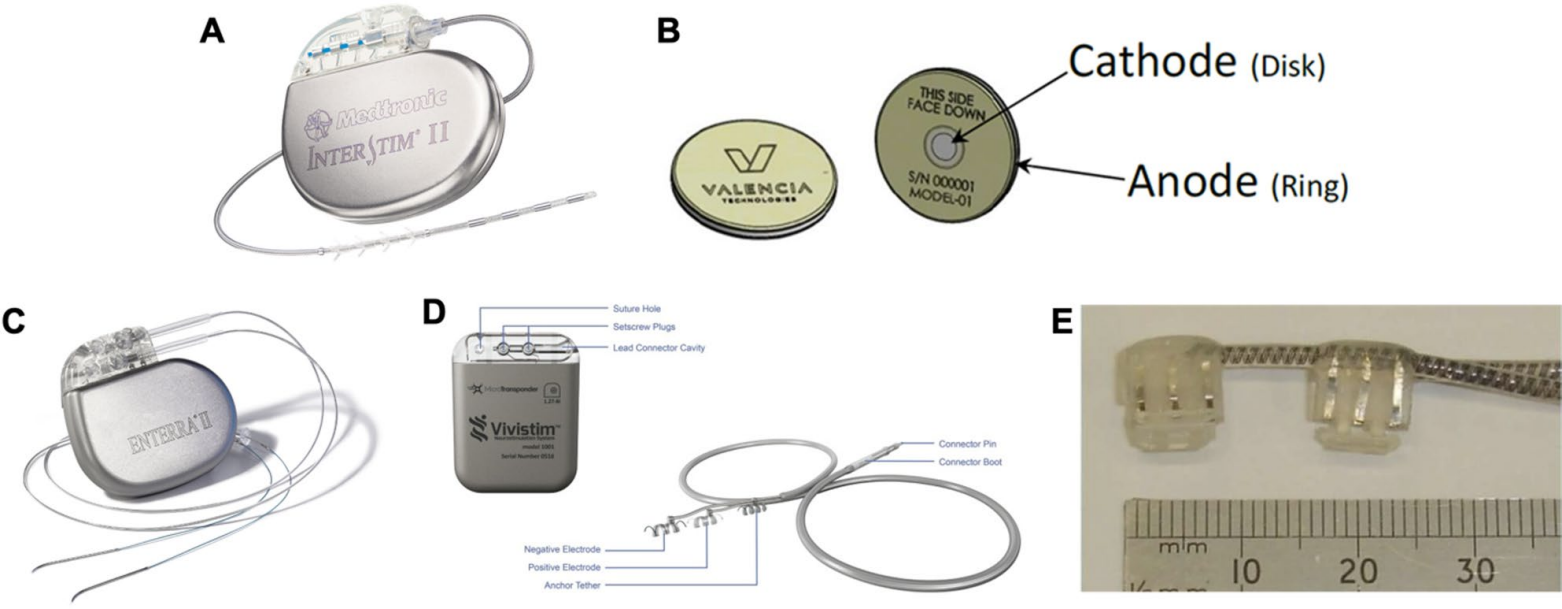
FDA approved devices illustrating different types of peripheral nerve interfaces. (**A**) Medtronic, Inc. InterStim™ II System for bladder and bowel control featuring electrode sites distributed along a flexible lead having anchoring tines. Copyright 2025 Medtronic. (**B**) Schematic illustrations of the Valencia Technologies Corp. eCoin^®^ Peripheral Neurostimulator for bladder control, featuring a leadless design where electrodes and electrical components are integrated into a single body. Copyright 2021 Valencia Technologies. (**C**) Enterra Medical, Inc. Enterra^®^ II Neurostimulator system with wire-like leads threaded into tissue to interface with nearby nerve targets. Copyright 2025 Enterra Medical, Inc. (**D**) Microtransponder Inc. Vivistim^®^ Paired VNS™ System for vagus nerve stimulation via helical cuff electrodes and an implantable pulse generator. Adapted with permission from (Liu et al. 2022). Copyright Elsevier 2022. (**E**) NeuroControl Corporation VOCARE Bladder System’s sacral anterior root stimulation electrodes used for bladder control. Adapted from (Yildiz et al. 2020) under CC BY 4.0

Peripheral nerve stimulation therapies that have gained FDA-approval (Table 2) generally favor a few form factors. The electrode-based PNI may be attached to an implantable pulse generator through a flexible lead, similar to traditional cardiac pacemakers, or have a leadless design in which the electronics and interface are integrated into a single, compact implanted unit (Fig. 3).

When greater isolation of the stimulus in relation to the nerve is required, extraneural cuff electrodes can be used. The electrodes in these cuffs are embedded in a tubular insulating structure that wraps around the nerve circumference, maintaining proximity and providing some signal isolation; many variations of this basic form factor are available, from electrodes shaped in multiple helical turns for improving contact area to cuffs having radially distributed disc electrodes (Hoffer and Loeb 1980; LivaNova 2021; MicroTransponder Inc, n.d.) (Fig. 3D). To gain further access to the nerve, the Finetech-Brindley electrode, developed in the 1970s, has three or five molded silicone channels into which individual sacral roots could be placed adjacent to embedded platinum foil electrodes that direct stimulation to empty the bladder and bowel in spinal cord injury patients (Brindley 1977) (Fig. 3E).

Likewise, the composition of clinical PNIs in FDA approved devices has converged towards a few common materials. Most electrode leads or cuffs use either medical-grade silicone or polyurethane as insulation material, which are applied using molding and extrusion processes. Wiring consists of coiled stainless steel, MP35N, or platinum alloys to prevent discontinuity when pulled (Wilks et al. 2017). Electrode sites that interface directly with tissue are typically constructed using bulk metal sources and fabricated with conventional machining (e.g., punched metal foils 5–25 μm thick embedded in ~ 40–500 μm thick silicone rubber) (Ordonez et al. 2012). For stimulation, platinum-iridium alloys or sputtered iridium oxide films (SIROF) on electrodes are often selected for their charge injection properties whereas stainless steel and MP35N, which are more cost effective, may be used in recording (*CVRx | Barostim™ | Heart Failure Device Implant*, n.d.).

Although there are many nerve targets of interest having diameters near or below 1 mm, these dimensions are at the limit of what is achievable with conventional construction methods for cuffs. Current FDA-approved devices with cuff style PNIs are compatible with nerves having diameters larger than 2 mm (Dougherty et al. 2023). However, there are many reports of novel approaches to selective PNIs in the research literature, with some that have progressed to human testing under investigational device exemption (IDE) (Hutchinson 2022; Kundu et al. 2014). In the next section, design considerations to achieve smaller PNIs for nerves having diameters near or below 1 mm are discussed followed by a review of candidate materials and the different approaches reported.

## Design considerations and materials for small peripheral nerve interfaces

**Fig. 4:**
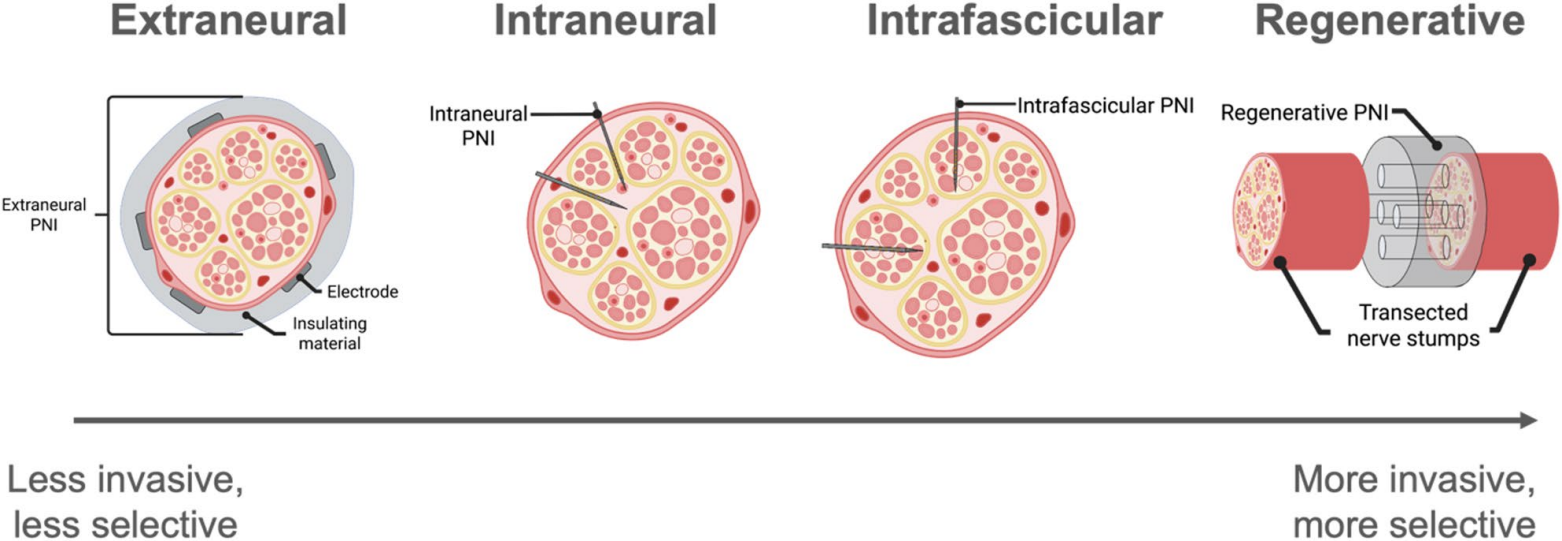
Diagram of common PNI formats and their respective invasiveness. Created in BioRender. (Li 2026) https://BioRender.com/ax8r1mx

Generally, placing an electrode as close as possible to the signal source is desirable for both stimulation and recording, enabling reduced power consumption and increased selectivity or resolution (Grill and Mortimer 1994; Loeb and Peck 1996; Tyler and Durand 2002). However, the anatomical structure of peripheral nerves (introduced in Fig. 2) and the surrounding tissue in which they are embedded present challenges related to foreign body reactions (FBR) and electrode-tissue interface stability that dictate necessary tradeoffs in interface design. PNIs can be categorized by the electrode placement relative to the nerve fiber: extraneural (electrode external to epineurium), intraneural (electrode internal to the epineurium but not the fascicles), intrafascicular (electrode within individual fascicle), and regenerative (electrode contained within bisected, regenerating nerve). The degree of invasiveness and injury to the nerve correlates to the achievable selectivity and resolution (Fig. 4). The approved devices in Table 2 are all classified as extraneural.

**Table 2:** FDA-approved devices with peripheral neural interfaces

Device Name/Manufacturer	Nerve Target (diameter in mm)	Interface Form Factor	Approval Year	Approval Indication	Reference
Barostim NEO System/CVRx	Carotid sinus nerve	2 mm diameter PtIr wire with IrOx coating (Monopolar Electrode)	2019	Improvement of heart failure symptoms and quality of life	(Food and Drug Administration 2019)
Proclaim DRG System/Abbott	Dorsal root ganglion (1–2)	Cylindrical multi-electrode (1.25 mm contacts) leads	2016	Complex regional pain syndrome (CRPS) Types I and II	(Food and Drug Administration 2016b)
Enterra II System/Medtronic	Gastric wall of antrum (N/A)	Cylindrical electrode with trumpet anchor	2024	Gastroparesis	(Food and Drug Administration 2016a)
Inspire Sleep Apnea Device/Inspire Medical Systems	Hypoglossal nerve (2–3)	Self-sizing cuff electrode	2014	Obstructive sleep apnea	(Food and Drug Administration 2014)
Genio Hypoglossal Nerve Stimulation System/Nyxoah	Hypoglossal nerves (2–3)	Two paddles each with two surface electrodes	2025	Obstructive sleep apnea for patients who failed/refused CPAP	(Food and Drug Administration 2025)
Avery Diaphragm Pacemaker/Avery Biomedical	Phrenic nerves (2–4)	Cuff electrode	1987	Respiratory insufficiency	(Food and Drug Administration 1987)
NeuRx Diaphragm Pacing/Synapse Biomedical	Phrenic nerves (2–4)	Percutaneous intramuscular diaphragm electrodes	2023	Respiratory insufficiency	(Food and Drug Administration 2023b)
InterStim System/Medtronic	Sacral nerves (3–5)	Self anchoring cylindrical multi-electrode lead	1998	Overactive bladder, urinary retention, fecal incontinence	(Food and Drug Administration 1997)
Vocare Bladder System/Neurocontrol	Sacral anterior/posterior roots (4–6)	Extradural surface electrodes	1998	Bladder control in complete Spinal Cord Injury	(Food and Drug Administration 1998)
Axonics Sacral Neuromodulation System/Axonics/Boston Scientific	Sacral nerves (3–5)	Self anchoring cylindrical multi-electrode lead	2019	Overactive bladder, fecal incontinence, urinary retention	(Food and Drug Administration 2019)
Neuspera Integrated SNM (iSNM) System/Neuspera Medical	Sacral nerves (3–5)	Wireless self anchoring cylindrical multi-electrode lead	2025	Urinary urge incontinence	(Food and Drug Administration 2025)
eCoin Peripheral Neurostimulator/Valencia Technologies	Tibial nerve (3–4)	Wireless implantable stimulator	2022	Overactive bladder symptoms	(Food and Drug Administration 2022)
Revi Tibial Neuromodulation System/BlueWind Medical	Tibial nerve (3–4)	Wireless implantable stimulator	2023	Urge urinary incontinence (UUI)	(Food and Drug Administration 2023a)
LivaNova VNS Therapy/LivaNova	Vagus nerve (2–3)	Helical cuff electrode	1997	Refractory epilepsy, treatment-resistant depression	(Food and Drug Administration 1997)
Vivistim Paired VNS System/MicroTransponder	Vagus nerve (2–3)	Helical cuff electrode	2021	Stroke rehabilitation (upper limb motor recovery)	(Food and Drug Administration 2021b)
StimRouter System/Bioness	Various peripheral nerves (1–8)	Wireless self anchoring cylindrical multi-electrode lead	2015	Chronic pain	(Food and Drug Administration 2015)
Neuspera Ultra-Miniaturized System/Neuspera Medical	Various peripheral nerves (1–8)	Wireless self anchoring cylindrical multi-electrode lead	2021	Chronic pain	(Food and Drug Administration 2021a)
Altius Direct Nerve Stimulation System/Neuros Medical	Residual limb nerves (2–6)	Cuff electrode	2024	Phantom limb pain and residual limb pain in leg amputees	(Food and Drug Administration 2024c)
Freedom PNS System/Curonix	Various peripheral nerves (1–8)	Self anchoring cylindrical multi-electrode lead	2024	Intractable pain of peripheral nerve	(Food and Drug Administration 2024a)
Nalu Neurostimulation System/Nalu Medical	Various peripheral nerves (1–8)	Cylindrical multi-electrode lead	2024	Intractable pain of peripheral nerve	(Food and Drug Administration 2024b)

Interfacing with smaller peripheral nerve branches imposes complex requirements on PNI construction. PNIs consist of a minimum of two materials to form the insulating and conductive regions, with the latter including the electrode sites, traces, and interconnections that connect to recording or stimulating electronics. Materials must accommodate small nerve diameters, providing close positioning of the electrode to the nerve and adequate insulation to prevent signal leakage. The PNI should not apply excessive pressure that constricts the nerve but should remain in place, as well as maintain its integrity, for the duration of use. Although minimizing the overall footprint of the PNI is desirable to prevent substantial tissue displacement and adverse immune responses, mechanical and electrical performance demand minimum layer thicknesses to provide adequate moisture barrier properties or low trace resistance, respectively. In addition, materials selected to construct PNIs should ideally be as soft as possible to more closely match the mechanical stiffness, or Young’s modulus, of the nerve and adjacent tissues to minimize the immune response to implantation.

Ideally, the PNI will be easy to surgically deploy and, if it is invasive, cause minimal damage. Nerves in high-motion areas are arranged to allow sliding and rearrangement (Millesi et al. 1995), imposing additional mechanical constraints on PNIs intended for deployment in such regions (Sunderland 1990). The materials selected and the overall design should avoid the potential for chronic irritation at the tissue-device interface and damage to adjacent tissues caused by the presence of the implanted interface. In addition to the bulk properties of materials used in PNIs, the implant’s surface chemistry should enable productive electrochemical interactions at electrode sites and avoid adverse immune responses at either conductive or insulating interfaces (Anderson et al. 2008).

To achieve miniaturized interfaces suitable for smaller nerves, materials that retain high electrical and mechanical performance, while also supporting thin film formats and processing techniques, are preferred. Importantly, thinner and more compliant materials are generally selected to reduce mechanical mismatch with tissue properties. Altogether, this entails incorporating techniques borrowed from or inspired by semiconductor manufacturing, novel three-dimensional printing techniques, and other methods adapted to achieve fine features with high spatial resolution. Insulating and conductive materials that support thin film and miniaturized PNI construction are briefly reviewed and discussed here.

### Insulating materials

Insulating materials are used to encapsulate and protect the conductive features of a PNI from the body’s harsh environment. In addition, their surface area dominates the interface formed with the body, playing a role in the elicited immune response after implantation in the body. This immune response can in turn trigger the production of reactive oxygen species, initiating a cascade of oxidative events which may lead to interlayer delamination and moisture penetration into the conductive traces, ultimately resulting in device failure (Kuliasha and Judy 2018; Takmakov 2017). Table 3 lists select material properties of common polymers used as insulating materials in PNIs, which are further discussed below (Table 4).

**Table 3:** A comparison of select material properties of polymers commonly used as insulation in nerve interfaces

**Material**	**Tensile Strength (MPa)**	**Young’s Modulus (GPa)**	**Water Absorption (%)**	**Dielectric Constant (MHz)**
PDMS^i^	6.2	0.1–0.5	< 1.0	2.6
Parylene C^i^	69	3.2	0.06–0.6	3
Polyimide^i^	128	2.4	2.8	3.3
LCPs^ii^	270–500	2–10	< 0.04	2.9
SMPs^iii^	0–25	130–400	3–8	< 3

i (Rihani et al., 2022)

ii (Rihani et al. 2022; Rowan et al. 2022)

iii (He et al. 2020; Ohki et al. 2004; Yu et al. 2011)

**Table 4:** Comparison and summary of insulating materials

**Material**	**Summary**
PDMS	A type of silicone rubber typically processed by casting that offers tunable mechanical properties, provides electrical insulation, and has been widely used in PNIs.
Parylene C	A transparent, thin film, thermoplastic polymer that is vapor deposited, compatible with microfabrication, can be thermoformed into 3-dimensional structures, and is widely used in PNIs.
Polyimide	A thin film polymer available in different formulations that is spin coated then cured, compatible with microfabrication, can sometimes be thermoformed, and is widely used in PNIs; certain formulations allow thermforming.
LCPs	A class of materials in the crystal phase with superior resistance against water absorption available as thicker films that require thermal processing to create multi-layer devices and may be promising for PNIs.
SMPs	An emerging class of polymer used for PNIs that change shape or soften due to external stimuli to reduce the mechanical mismatch between the tissue and device and are compatible with microfabrication.

#### Polydimethylsiloxanes

Early PNIs benefitted from the introduction of medical grade silicone rubbers in the 1960s, and particularly polydimethylsiloxanes (PDMS), to provide electrical insulation and structural support for metal wires and foils (Stein et al. 1977). PDMS comes in many formulations, and commonly used versions involve mixing a prepolymer with a curing agent then casting into the final desired form. The resulting Young’s modulus of the cured polymer is adjustable (0.4 kPa to 40 MPa) by changing the ratio of curing agent to prepolymer and modifying processing parameters such as the curing temperature (Niemiec and Kim 2024). It is also possible to use semiconductor manufacturing techniques to spin coat thin layers of PDMS on a flat substrate (Bračič et al. 2014), achieving thicknesses from sub-micron to hundreds of microns (Chen et al. 2012). Using non-medical grade silicones, laser-patterning and embossing were demonstrated and used to produce PNI features (Paggi et al. 2024; Terkan et al. 2020). Similarly, a network of microchannels can be imprinted into PDMS using micromolding to produce a scaffold for nerve regeneration (Choi et al. 2014; Kim et al. 2014a, b). Although PDMS has a low Young’s modulus compared to other polymers, making it a popular choice for soft, stretchable electronics, thicker layers are generally required to avoid breakage due to its low tensile strength^1^. Therefore, it difficult to use this material for constructing smaller nerve interfaces, especially below 1 mm in diameter (Lienemann et al. 2023; Paggi et al. 2024).

#### Parylene C

Poly-para-xylylene, more commonly known by the trade name Parylene, is a class of polymers applied as a thin, conformal coating. The Parylene C variant is the most widely used for biomedical applications, and like other Parylenes, is deposited using chemical vapor deposition, during which a solid, granular precursor in dimer form is vaporized into a gaseous monomer that then polymerizes on exposed surfaces, typically at room temperature, creating a conformal and pinhole-free polymer coating (Liu et al. 2018). Layer thicknesses range from 0.1 to several tens of microns. Thicker Parylene C layers generally offer superior dielectric properties and chemical and mechanical protection. Interlayer adhesion between Parylene and Parylene-metal layers may require adhesion promoting strategies and thermal treatment when processed by standard microfabrication techniques (Ortigoza-Diaz et al. 2018). Although microfabrication typically results in planar structures, three-dimensional structures have been reported using post-microfabrication modification of the thermoplastic via thermoforming, which involves fixturing films into the desired shape and then heating above the glass transition temperature. This induces the amorphous regions to reorganize, locking in the new shape. Such modification enabled small diameter helices down to 0.25 mm and the realization of nerve cuff structures (B. J. Kim et al. 2014; Thielen and Meng 2023). New developments have shown that stretchability can be obtained by introducing nanoscale wrinkles (Huang et al. 2025).

#### Polyimide

Polyimide is a class of polymers compatible with microfabrication and hence is also commonly used for insulation of PNIs. Although available in sheet and film formats, polyimide typically comes in a liquid formulation as a precursor dissolved in solvents, such as dimethyl sulfoxide and tetrahydro-furan, and is applied via spin coating. Layer thicknesses typically range from 5 to 80 μm (Kumar et al. 2023; Kullmann et al. 2022). Following a series of baking steps, imidization is induced to form the final polymer and subsequent microfabrication steps can be performed. Polyimide films can also be thermoformed to create three dimensional structures (Verker et al. 2016; Sun et al. 2023). Interlayer adhesion between polyimide layers and polyimide-to-metal may require adhesion promoting strategies (Lee et al. 2016; Cen-Puc et al. 2021; Kuliasha et al. 2018).

#### Liquid crystal polymers

Liquid crystal polymers (LCPs) are a class of thermoplastics that take advantage of the liquid crystal phase to achieve unique mechanical properties (Rihani et al. 2022). The material exhibits low water absorption in comparison to other common polymers, potentially leading to better long-term robustness in vivo. LCPs can be processed by laser cutting alignment marks onto commercially available films and thermally pressing for adhesion. Typical layer thicknesses range from 25 to 50 μm (Jeong et al. 2012, 2019; Yun et al. 2022). LCP-based implants can be thermoformed with metal fixtures using heat and pressure (Mogilevsky et al. 1998). Unlike other polymers that rely on chemical and mechanical surface modifications to promote interlayer adhesion, LCP layers can be joined more effectively by using formulations with different melting points. LCP films with low melting temperatures can be used as bonding layers between LCP films with higher melting temperatures. The adhesion between LCP layers has been shown to be stronger in comparison with polyimides and Parylene C when subject to heated in vitro conditions (Jeong et al. 2012, 2016). Although LCPs have been used more extensively in brain neural interfaces, its potential for PNIs has also been demonstrated in a flat interface nerve electrode (FINE) (Hess et al. 2007).

#### Shape memory polymers

Shape memory polymers (SMPs) change their shape in response to external stimuli, such as thermal stress and mechanical deformation (González-González et al. 2018). In addition, the Young’s modulus of SMPs can be adjusted by external stimuli including exposure to aqueous environments or changes in temperature. This feature offers an advantage over other materials in that SMP-based interfaces can exhibit an initially high stiffness during implantation that reduces in response to temperature and moisture to match tissue properties and minimize the fibrotic response (Dai et al. 2025). SMP-based nerve interfaces can be manufactured through existing photolithographic processes, with the SMP layers themselves either being cast and cured or spin coated (González-González et al. 2018). Typical layer thicknesses are upwards of 20 μm (Cho et al. 2021). SMP was used to produce small nerve interfaces for rat pelvic and sciatic nerves to allow softening to reduce the mechanical mismatch between device-tissue properties and enable a closer interface fit around the nerve using the shape memory effect (Cho et al. 2021; González-González et al. 2018).

### Conductive materials

The conductive materials used in a PNI serve multiple purposes including contact pads, conductive traces, and the electrode interface, all of which are connected together to create a continuous electrical conduit between the tissue and externalized or implanted instrumentation used for measurement or stimulation. Each of these features may have a distinct composition or be formed from the same material. As such, the material or material chain should have low resistance, or high conductance, and withstand any mechanical motion as it interacts with adjacent tissues, although the actual electrode-tissue interface should ideally be stationary. Early PNIs commonly used bulk Pt or Pt alloy wires and foils embedded in silicone (Gablech and Głowacki 2023). While bulk materials may still be used between the actual PNI and instrumentation, especially if there are long distances between them, to achieve a small scale and low profile PNI, thin film materials such as Au, Pt, and Ti may be preferred to create the electrodes and traces (Cordill et al., 2022). Structured films such as microcracked Au and novel conductors such as conductive polymers have also been explored. These conductive structural materials and coatings for modifying the electrochemical properties of the exposed electrode sites are briefly reviewed below (Table 5).

**Table 5:** Comparison and summary of conductive materials

**Material**	**Summary**
Thin film metals	Examples such as Au and Pt provide excellent electrical conductivity, are compatible with microfabrication processes, and can be deposited in nanometer scale thicknesses and patterned into features with micron scale precision.
Liquid metals	Capable of maintaining electrical continuity under mechanical deformation but are limited in achievable dimensions and are typically patterned using microchannels.
Doped conductors	Mixtures of a flexible nonconductive material and a conductive dopant to achieve flexible conductors with reduced stiffness.
Conductive polymers	Examples such as PEDOT and polypyrrole can be produced in thin films, often with tunable properties, and may offer transparency or MRI compatibility.
Conductive hydrogels	Consist of a tunable hydrophilic polymer doped with conducting ions to serve as a flexible and elastic conduit for current delivery.

#### Thin film metal conductors

Thin film metals, typically deposited using thermal evaporation, electron beam evaporation, or sputtering, can be hundreds of nanometers thick and popular choices include Au, Pt, Ti, or Ir. Since Au and Pt can exhibit poor adhesion, a thin adhesion layer, such as Ti, may be used, resulting in a multi-metal stack (Gablech and Głowacki 2023). To manage residual stress in the metal layer, which may induce undesirable curling or even cracking, additional layers of different metals may be added (Huff 2022; Scholten et al. 2025). Diffusion barriers of platinum prevent alloying of adjacent metal layers if there are device fabrication processes involving high temperatures in later stages (Kuliasha and Judy 2021; Poate 1981).

Although conductive features are usually formed from continuous films, depositing metals on intentionally roughened surfaces can produce conductive films with microcracks. As the cracks are distributed randomly, there exist connected islands of conductivity that tolerate greater mechanical strain experienced during natural stretching and bending in vivo, preventing failures in trace continuity or delamination exhibited in continuous thin film metal features. For example, compared to uncracked thin film Ti/Au, microcracked Ti/Au retains electrical conductivity and gains tissue-conforming mechanical properties (Hiendlmeier et al. 2023).

#### Liquid metals and their alloys

The fragility of lengthy thin film metal traces has prompted investigation of alternative materials that are robust to strain and bending stress. Liquid metals (LMs), such as gallium, and their alloys, such as eutectic gallium-indium (EGaIn), are liquid near room temperature and when contained in silicone microchannels, can serve as conductors (Zhang et al. 2024; Tang et al. 2022). Wafer-level batch processes like printing, syringe injection, selective wetting, and layer by layer deposition can be used to apply LM-based conductors to substrates (Zhang et al. 2024). However, LMs are generally applied in larger volumes relative to thin film traces which limits scaling of feature sizes. Ga-based LMs oxidize in contact with aqueous environments and therefore are unsuitable for the electrode interface (Lim et al., 2022).

#### Doped conductors

Flexible nonconductive materials can be mixed with dopants to produce conductors that adopt the electrical properties of the dopant and the mechanical properties of the substrate, enabling tunable combinations of electrical conductivities and Young’s moduli to address the mechanical mismatch between the device and tissue. In addition, such composites can tolerate greater mechanical deformations than thin film conductors. For example, carbon-based conductors such as graphene, carbon nanotubes (CNT), and graphite all leverage a specific hybridized form of the carbon atom to conduct current. When mixed into elastomers such as PDMS, flexible conductors can be screen printed and incorporated into interfaces for small-diameter nerves (Montoya et al. 2023; Terkan et al. 2020) MXenes, 2D transition metal carbides of the chemical formula Ti_3_C_2_T_x_, have high conductivity in coated fiber electrodes (Bi et al. 2024). Their flake-like physical form can be mixed into liquid elastomers to produce flexible conductors. Poly(3,4-ethylenedioxythiophene): polystyrene sulfonate (PEDOT:PSS) has also been used as a dopant in polyurethane in nerve cuffs (Cuttaz et al. 2021), and hydrogel in microelectrode arrays (Won et al. 2022, 2024).

#### Polypyrrole

Polypyrrole (PPy) is a conductive polymer that is formed by the polymerization of pyrrole, an aromatic organic compound. PPy can be deposited via chemical oxidation polymerization where the monomer is polymerized with the aid of a doping agent and an oxidizing agent (Sevcencu et al. 2025). In addition, pyrrole can be electropolymerized via chronopotentiometry (Luo and Cui 2009). PPy exhibits many properties (i.e., biocompatibility, chemical stability, high conductivity) that make it an intriguing electrode coating material. However, PPy has limited long-term electrochemical stability due to defect sites along its polymer chain structure that are thought to be the origin of over-oxidative breakdown (Cui and Martin 2003; Yamato et al. 1995). Despite attempts to improve its properties by incorporating multi-walled carbon nanotubes (Green et al. 2008), graphene oxide (Deng et al. 2011), or naphthalin-2-sulfonic acid (Sevcencu et al. 2025), PPy has seen limited adoption compared to other conductive polymers.

#### Poly(3,4-ethylenedioxythiophene)

Poly(3,4-ethylenedioxythiophene) (PEDOT) is a conductive polymer that is usually doped with poly-polystyrene sulfonate (PSS) and has been used as a replacement for metals in neural interfaces in applications where device transparency or compatibility with magnetic resonance imaging (MRI) is required (Kim et al. 2025). The excellent solution dispersibility allows the material to be applied by spin coating (typically 400 nm thick) (Dijk et al. 2022; Middya et al. 2025) Additionally, the material properties are tunable by incorporating polymers and conductive fillers with the desired electrical and mechanical (such as viscosity, elasticity, or shear-thinning) properties (Li et al. 2025a, b; Ruiz-Mateos Serrano et al. 2024).

#### Conductive hydrogels

Hydrogels are hydrophilic polymers that have a tunable mechanical modulus based on the weight of hydrogel polymer that is mixed into water, which can be adjusted to mimic neural tissue. As such, hydrogels can provide a buffer layer against soft tissue when used as an encapsulating coating for rigid electrodes, reducing the foreign body response (FBR) to implantation. Hydrogels can become conductive when doped with conducting ions such as salts (Thakur et al. 2021), or after conductive polymers are synthesized into the hydrogel matrix or crosslinked with the hydrogel polymer (Fu et al. 2020). Homogeneous integration of the conductive polymers into the hydrogel matrix avoids formation of stratified composites which have poor cohesion (Green et al. 2012). Water-soluble drugs and growth factors can be incorporated into the polymer to minimize inflammation and FBR or promote neuron survival (Duan et al. 2024). Conductive hydrogels can be used to fill the gap between cuff electrodes and the nerve (Yang et al. 2024). In addition, hydrogels can provide separation between metal electrodes and the nerve for stimulation regimes involving long-duration pulses or direct current to mitigate irreversible electrochemical processes (Ackermann et al. 2011; Thakur et al. 2021) (Tables 6 and 7).

**Table 6:** A comparison of electrode surface modification materials and methods *vs.* unmodified platinum

**Material**	**Fabrication method**	Maximum CIC [mC/cm^2^]
Bare Pt	Evaporation, sputtering^i^	0.1
	Foil^ii^	0.015 @ 0.5 ms
Roughened Pt	Electrochemical roughening^iii^	1.0
	Laser ablation^iv^	0.15 @ 0.8 ms
	Plasma (dry) etching^v^	0.8
TiN	Reactive sputtering^vi^	1
PtIr	Electrodeposition^vii^	0.055
IrOx	Electrodeposition (EIROF)^viii^	1.2
	Reactive sputtering (SIROF)^ix^	5
	Electrochemical activation (AIROF)^x^	4 @ 0.2 ms
RuOx	Reactive sputtering^xi^	7.1 @ 0.2 ms
Carbon Nanotubes	Low pressure chemical vapor deposition^xii^	1.0–1.6
Porous (Activated) Graphene	Laser reduction^xiii^	3.1
	Electrochemical reduction^xiv^	0.07
PEDOT:PSS	Electrodeposition^x^	15

i (Rezard et al. 2025)

ii (Cisnal et al. 2019)

iii (Weremfo et al. 2015)

iv (Green et al. 2014)

v (Leber et al. 2017)

vi (Cogan 2008)

vii (Dalrymple et al. 2019)

viii (Iwasa et al. 2020)

ix (Cogan 2008)

x (Weiland et al. 2002)

xi (Chakraborty et al. 2022)

xii (Iwasa et al. 2020)

xiii (Iwasa et al. 2020)

xiv (Dalrymple et al. 2019)

xv (Cogan 2008)

**Table 7:** Comparison and summary of electrode coatings

**Material**	**Summary**
TiN	Rough and porous morphology increases electrochemical surface area, deposition process compatible with batch microfabrication techniques, performance limited by restricted electrolyte access to deeper pores.
PtIr	Improves electrochemical properties by combining ductile nature of Pt and charge transfer properties of iridium, applied by electrodeposition or sputtering.
IrOx	Improves electrochemical properties through multiple valence oxides of iridium, applied via different processes that give rise to different variations (AIROF, EIROF, and SIROF).
RuOx	Improves electrochemical properties through nodular morphology, applied by sputtering, more affordable than iridium-based coatings.
Carbon based materials	Increases surface area with potential to be highly conductive, cytotoxicity properties still under investigation.
PEDOT:PSS	Comparable performance to metal based coatings but may exhibit delamination under long term use.

### Electrode coatings

**Fig. 5:**
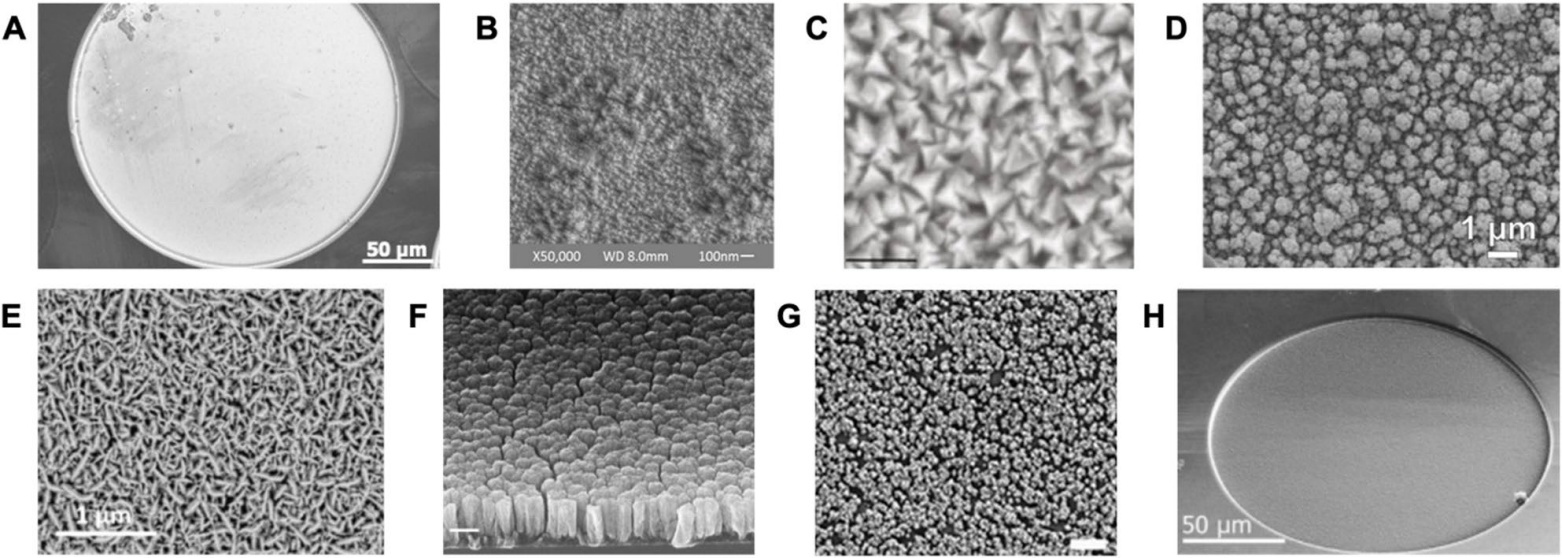
Scanning electron microscope (SEM) images of electrode surfaces, including those with additional modifications to improve electrochemical performance. (**A)** Electron beam evaporated Pt electrode. For fabrication methods, see (Rezard et al. 2025). (**B**) Pt electrode after electrochemical roughening in sulfuric acid, resulting in a 21× area increase. Adapted from (Ivanovskaya et al. 2018) under CC BY NC ND 4.0. (**C**) TiN coating with fractal nanostructures (scale bar = 100 nm). Reproduced from (González-González et al. 2018) under CC BY 4.0. (**D)** Electroplated 60 − 40% Pt-Ir coating. Reproduced from (Baldwin et al. 2018). Copyright IEEE 2018. (**E)** IrOx layer deposited by reactive sputtering. Reproduced from (Sun et al. 2023) under CC BY NC ND 4.0. (**F)** Sputtered RuOx on cleaved silicon wafer (scale bar = 200 nm). Reproduced with permission from (Atmaramani et al. 2020). Copyright Elsevier 2020. (**G)** Platinum nanoparticle coating on graphene via chemical vapor deposition (scale bar = 1 μm). Reproduced from (Doğan and Bayram 2025) under CC BY NC 3.0. (**H**) Electrodeposited PEDOT:PSS coating on flexible Parylene C cuff electrode. Reproduced with permission from (Rezard et al. 2025). Copyright 2025 IEEE.

Electrode sites should have low electrochemical impedance^2^ for recording applications, and high charge storage capacity (CSC)^3^ and high charge injection capacity (CIC)^4^ for stimulation (Cogan 2008; Larson and Meng 2019; Merrill et al. 2005)^5^. To achieve such properties, smooth metallic electrode sites produced using thin film deposition methods may require additional surface modification such as the selective application of coatings. Surface modification may simply entail improving the roughness or porosity of the electrode site. For example, electrochemical roughening of smooth platinum electrodes can be achieved by forming a platinum oxide layer on the surface and later reducing it via repeated potential cycling (Fig. 5A-B) (Ivanovskaya et al. 2018; Weremfo et al. 2015) or electrodepositing a platinum-copper alloy followed by removing the copper (Boretius et al. 2011). Direct laser micromachining of an electrode surface can induce a pattern and hence restructuring (Amini et al. 2022; Blagojevic et al. 2024; Green et al. 2014). Roughening can also be achieved via mild plasma etching (Chung et al. 2015). However, the thickness of a thin film metal layer may need to be increased to survive more aggressive roughening techniques. Selective application of coatings, discussed below, may be applied to electrode sites, either all at once in a wafer level process, one device at a time, or one site at a time. Rough surfaces, however, may be prone to fouling since they provide more surface area for absorption of nonconductive biomaterial (Ding et al. 2022). Additionally, conductive polymer coatings not only enhance electrode performance by allowing capacitive charge transfer processes to occur, but also act as a physical barrier to protect metal electrodes from corrosion due to oxygen reduction in the presence of reactive oxygen species and during the cathodic phase of the stimulation waveform, if the coating has strong adhesion and is defect-free *(*Duan et al. 2024; Paulsen et al. 2020; Venkatraman et al. 2011). A comparison of different materials used for improving electrochemical properties and the respective improvements in CIC are provided in Table 6.

#### Titanium nitride

Titanium nitride (TiN) coatings exhibit a porous and rough morphology which greatly increase the electrochemical surface area (Cogan 2008) and can be deposited via reactive sputtering with a titanium target and nitrogen gas, where stoichiometric properties can be tuned, or via atomic layer deposition with a dedicated titanium nitride target. For applications requiring higher current densities, TiN performance is limited by electrolyte access to pores, arising in a delay-line with a time constant. Therefore, shallow pores are preferred since narrow and deep pores will have higher time constants (Cogan 2008). TiN has been employed on highly flexible electrodes to interface with the pelvic nerve of rats (~ 200 μm) (Fig. 5C) (González-González et al. 2018).

#### Platinum iridium

The ductility of platinum and the charge transfer properties of iridium can be advantageously combined in PtIr coatings (Cassar et al. 2019; Dalrymple et al. 2019; Della Valle et al. 2021a; Ganske et al. 2011). An electrodeposited coating comprising of approximately 60% Pt and 40% Ir by molar fraction was reported to have improved surface area and recording and stimulation performance over bulk PtIr microwires (Cassar et al. 2019). Higher platinum content reduces the inherent capacitance of the iridium but compensates by increasing coating surface area. PtIr (~ 70:30) was electrodeposited on the tips of carbon fiber electrodes (8 μm diameter), improving impedance and CIC in comparison to bare carbon fiber (Della Valle et al. 2021). Electrophoretically deposited PtIr (90:10) reduced the impedance and increased the electrochemical surface area compared to Pt wire coated with Pt nanoparticles (Fig. 5D) (Baldwin et al., 2018; Ramesh et al. 2024). In addition, PtIr coatings can be obtained via sputtering where the atomic ratio can be tuned based on application (Ganske et al. 2011) (Table 6).

#### Iridium oxide

Iridium oxide (IrOx) has multiple structural forms, with the most stable being a rutile crystalline structure (Xu et al. 2023). Applied films increase electrochemical performance by rapid, reversible faradaic reactions involving reduction and oxidation between the ionic states (Cogan 2008). Activated IrOx films (AIROFs) can be formed by performing repeated potential cycling on iridium films until the desired thickness and charge storage capacity of the oxide layer is obtained (Frederick et al. 2020). Electrodeposited IrOx films (EIROFs) are formed on non-iridium metal substrates by potential sweeps and pulsing in iridium-containing solutions (Khalil et al. 2016). Sputtered IrOx films (SIROFs) are created by reactive direct current (DC) sputtering of an iridium target. A thermoformed polyimide cuff electrode (86 ± 12 μm diameter) with SIROF for the mouse vagus nerve was reported and no differences in electrochemical performance or delamination post-thermoforming were observed (Fig. 5E) (Sun et al. 2023). Additionally, previous work reported comparable mechanical properties between EIROF and AIROF. The primary difference between them is that Ir surfaces are not required for EIROF formation, making it possible to apply EIROF to different metal electrodes (Meyer et al. 2001). Other studies investigated and compared the electrochemical properties of different IrOx films (Lutz et al. 2025; Xiao-Yang Kang et al. 2014) (Table 7).

#### Ruthenium oxide

The use of ruthenium oxide (RuOx) is relatively new and the coating has not yet been applied to peripheral nerve interfaces. It offers a potential alternative, based on its reported electrochemical properties, to IrOx which can be difficult and costly to source. Like SIROF, RuOx can be deposited via DC reactive sputtering, and requires oxygen, water vapor, and argon. Controlling the oxygen and water vapor flow rates (1:3) can improve performance, decrease impedance, and increase CIC and CSC to a level comparable to SIROF and surpassing TiN. These improvements are attributed to the formation of compact nodular islands when water vapor is added, increasing porosity and reducing crystallinity which together increased the surface area (Chakraborty et al. 2022). Preliminary sub-chronic implanted studies indicate the coating is non-cytotoxic and can record from the cortex with high signal to noise ratio (Fig. 5F) (Abbott et al. 2025; Atmaramani et al. 2020).

#### Carbon-based coatings

Carbon nanotube and graphene-based coatings exhibit large surface area and high conductance, which reduces electrode impedance (Duan et al. 2024). Carbon-based coatings and their composites can be applied electrochemically (Keefer et al., 2008) or through chemical vapor deposition (Fig. 5G) (Doğan and Bayram 2025). The potential cytotoxicity of these materials requires further investigation as carbon-based electrode coatings can aggregate due to their hydrophobicity and many different CNT manifestations (i.e., particle size, agglomerate size, immersion medium) are still under investigation (Duan et al. 2024).

#### Poly(3,4-ethylenedioxythiophene): polystyrene sulfonate

Poly(3,4-ethylenedioxythiophene): polystyrene sulfonate (PEDOT:PSS) can be spin coated or electrodeposited as an electrode coating using voltage or current cycling or sweeping, producing a porous surface and improvement in impedance and CIC (Carnicer-Lombarte et al. 2024; Duan et al. 2024; Ludwig et al. 2011; Shin et al. 2024). Electrodeposited PEDOT:PSS was used to selectively coat electrodes in a Parylene-based nerve cuff, remaining intact after thermoforming the initially planar device into the cylindrical cuff (Fig. 5H) (Rezard et al. 2025). However, PEDOT:PSS coatings may exhibit poor adhesion, resulting in delamination over long term use (Lee et al. 2016). Though, depositing PEDOT:PSS on other coatings to roughen the electrode surface, such as IrOx and nanostructured platinum, has been shown to increase longevity and reduce delamination (Boehler et al. 2017).

## Approaches to small peripheral nerve interfaces

### Extraneural small nerve interfaces

Extraneural interfaces apply electrodes to the nerve exterior and have been widely explored in a variety of formats. Silicone cuff electrodes found in clinical devices are difficult to further miniaturize as the manual manufacturing processes (silicone molding with embedded platinum foils) and materials used do not scale well to cuffs for nerves measuring 1 mm in diameter or less. Microfabrication provides micron-scale precision and therefore enables extremely thin extraneural interfaces that can be accurately produced in a wide variety of formats and include several unique features. In addition, microfabrication enables production of many identical devices simultaneously as part of a batch process. However, the implantation of devices with a small footprint or those composed of softer materials may be more difficult than that of larger, more rigid devices.

#### Microwires

**Fig. 6:**
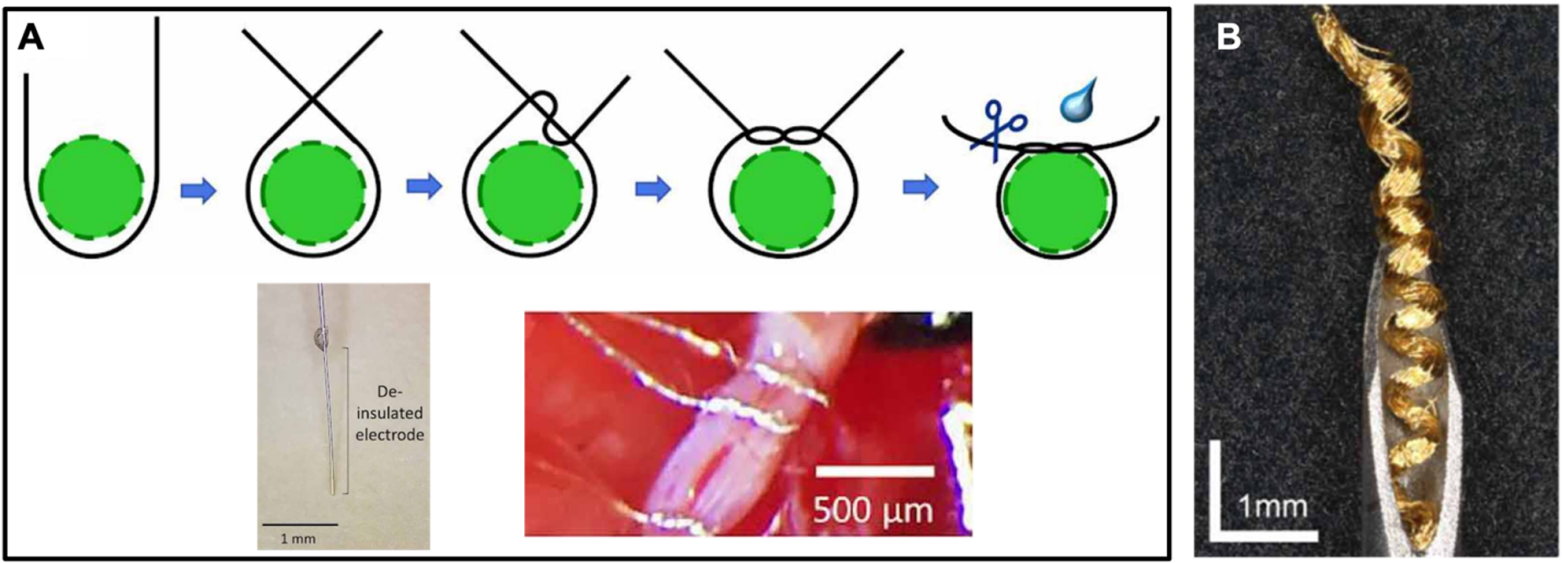
(**A)** Illustration in the top row shows microwire (black) tied in an overhand knot around the vagus nerve (green). In the final step, the excess wire is trimmed and Kwik-Sil (blue) is applied to the deinsulated wire. The bottom row shows photographs of the deinsulated portion of the microwire (scale bar = 1 mm) on the left and wrapped microwires around the cervical vagus nerve on the right. Adapted from (Falcone et al. 2020) with permission from IOP Publishing. Copyright 2020 IOP Publishing. (**B)** Gold helical microwire structure electrode. Adapted from (Lam et al. 2025) under CC BY 4.0.

The simplest interface to smaller nerves can be accomplished by wrapping conductive wires around the nerve. This technique was demonstrated on mouse cervical vagus nerve by surgically tying Teflon-insulated 12 μm Pt wires with deinsulated tips then applying silicone for electrical isolation (Fig. 6A) (Falcone et al. 2020). Wrapped wires could be placed closely together (~ 1 mm apart), were inexpensive, achieved comparable electrochemical performance to commercial cuffs, and obtained stable in vivo performance (up to 60 days). A helical electrode was also developed using Au microwire for stimulation of rat sciatic nerve (3 mm in length and 0.6 mm in outer diameter of helix) (Fig. 6B) (Lam et al. 2025). The helical design improved tissue anchoring, resulting in stable impedance and muscle activation threshold. While convenient and inexpensive, such approaches are not scalable. Recent advances in materials, however, have introduced an alternative microwire approach involving the in situ polymerization of an injected prepolymer, allowing electrodes to be formed near target nerves. An injected cuff was formed around rat brachial plexus and tetanic muscle contractions demonstrated (Trevathan et al. 2019).

#### Manually secured cuffs

**Fig. 7:**
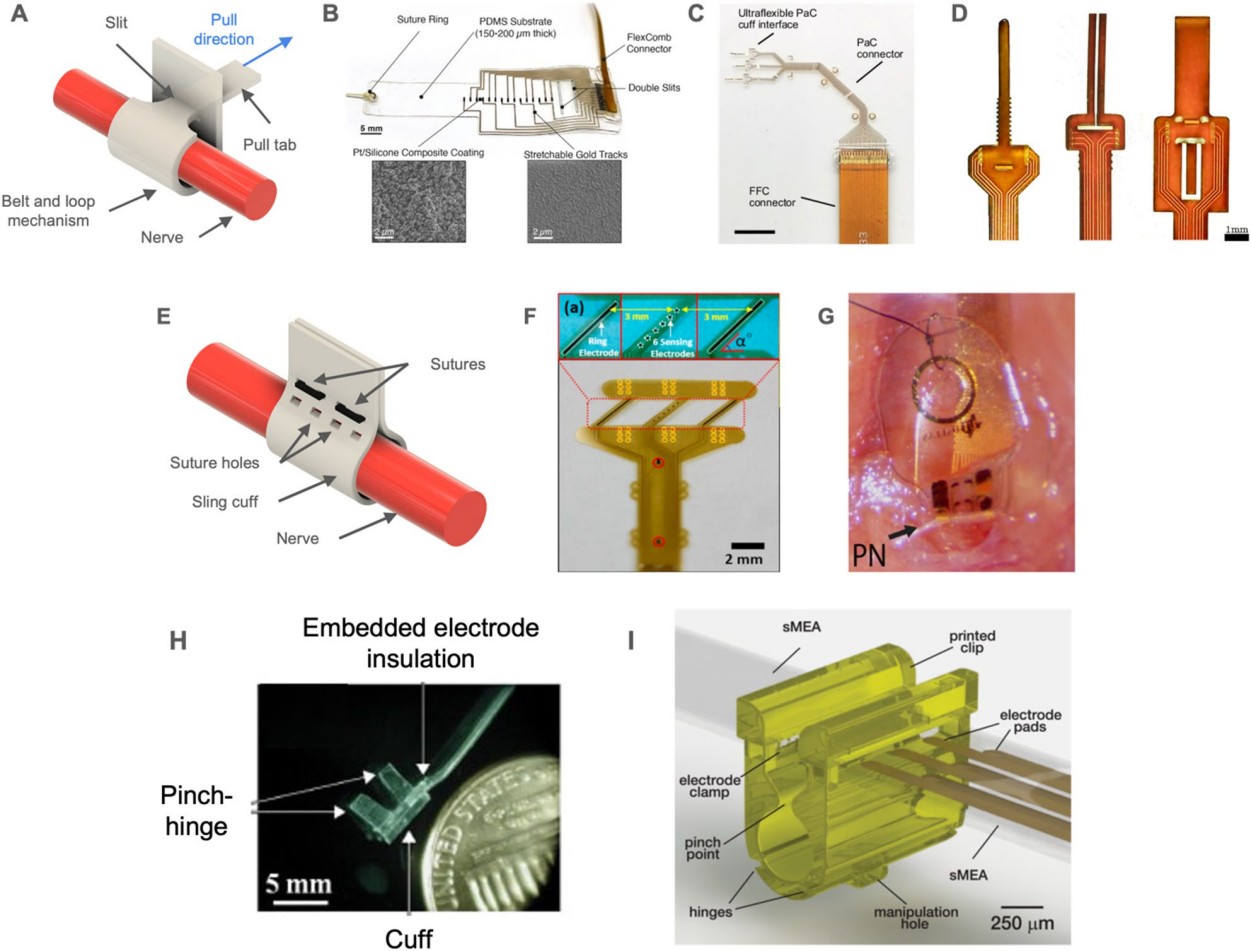
Examples of cuff electrodes that are manually secured. (**A**) Illustration of belt and loop locking mechanism concept. (**B**) A PDMS multi-contact cuff electrode and insets showing SEM images of electrode surface and traces. Reproduced from (Paggi et al. 2024) under CC BY 4.0. (**C**) A Parylene C cuff array designed to interface with the median, ulnar, and radial nerves of the rat forearm. Reproduced from (Carnicer-Lombarte et al. 2024) under CC BY 4.0. (**D**) Cuff electrodes fabricated using conventional flexible printed circuit technology. Adapted from (Riley et al. 2024) CC BY 4.0. (**E**) Illustration of sling cuff locking mechanism. (**F**) A polyimide sling electrode for the rat sciatic and peroneal nerves. Reprinted with permission from (S. Lee et al. 2017). Copyright 2017 Elsevier. (**G**) A shape memory polymer sling cuff implanted on the rat pelvic nerve (~200 µm diameter). Reproduced from (González-González et al. 2018) under CC BY 4.0. (**H**) A cuff electrode with an integrated pinch-hinge. Reproduced from (Korivi & Ajmera, 2011) with permission from Elsevier. Copyright 2011 Elsevier. (**I**) Rendering of a microclip with an integrated electrode array. The clip holds the nerve in close proximity to a flexible electrode array. Reproduced from (Otchy et al. 2020) under CC BY 4.0

A microfabricated cuff generally uses a thin-film polymer for structural support and electrical insulation of the conductive features that form the electrodes, traces, and contact pads. Since microfabrication entails the use of planar processes to produce layered structures, a cuff must incorporate a mechanism to wrap then secure it around the nerve. Wrapping can be performed manually followed by implementing a feature to secure the cuff in place relative to the nerve or the initially flat device can be curled and pre-formed such that it retains a cylindrical shape (Fig. 7).

A thin film PNI can be designed to include a tab and slit so that the tab can be threaded through the slit to wrap the device around the nerve (Fig. 7A-D). This design can accommodate nerves of different sizes and shapes and apply pressure uniformly to the nerve. A multi-contact device built on top of a silicone substrate (150 μm thick) was demonstrated acutely in pigs and rats that reduced fibrotic tissue formation in comparison to a commercial, fixed diameter silicone cuff (Paggi et al. 2024). Almost no fibrotic scarring was observed for a chronically implanted multi-contact Parylene C cuff (4 μm thick) on a bundle of radial, ulnar, and median nerves in rat when compared to commercial PDMS and polyethylene cuffs (Carnicer-Lombarte et al. 2024). Using flexible printed circuit technology, a multi-contact cuff electrode (78 μm thick) was realized for spatially selective stimulation and used a belt and loop mechanism (Jiman et al. 2020). Such devices can be produced rapidly and inexpensively by printed circuit board manufacturers.

Alternatively, a “sling” design in which the cuff ends are secured using standard sutures may reduce surgical handling of the nerve (Fig. 7E-G). The circumference of the nerve must be carefully considered, and variability in nerve diameter may be accounted for by having cuffs of different sizes available or by adjusting the fit using a single cuff with multiple suturing sites. A polyimide sling (12 μm thickness) used energy harvested from an integrated triboelectric nanogenerator to deliver stimulation to the rat sciatic and peroneal nerves (Lee et al. 2017). To improve the fit, thiol-ene/acrylate SMPs that soften from a Young’s modulus of 2,380 to 550 MPa near body temperature were incorporated into a device for the rat pelvic nerve (200 μm). When compared to commercial silicone cuffs, the softening sling was associated with reduced fibrotic tissue growth between the nerve and the cuff (Arreaga-Salas et al. 2015; González-González et al. 2018; Ware et al. 2012).

A rigid clip can be used to confine the nerve, holding it in place relative to an adjacent flexible electrode, without the need for additional sutures or adhesives (Fig. 7H-I) (Larson and Meng 2019). This concept was first introduced as a molded multi-layer silicone “pinch-hinge” cuff-like nerve enclosure with a pair of integrated metal wires (~ 300 μm inner diameter defined by a rod); pinching the integrated hinge opened the cuff to accept the nerve (Korivi and Ajmera 2011). A clip with various “trap door” mechanisms was reported in which two photon polymerization was used to produce the clip to retain a nerve next to integrated carbon nanotube fibers and in another iteration, microfabricated multielectrode arrays. Activity of the tracheal syringeal nerve (150 μm diameter) of zebra finches was recorded for 24 days using 6-electrode devices (Lissandrello et al. 2017; Rowan et al. 2022).

#### Self-closing

**Fig. 8:**
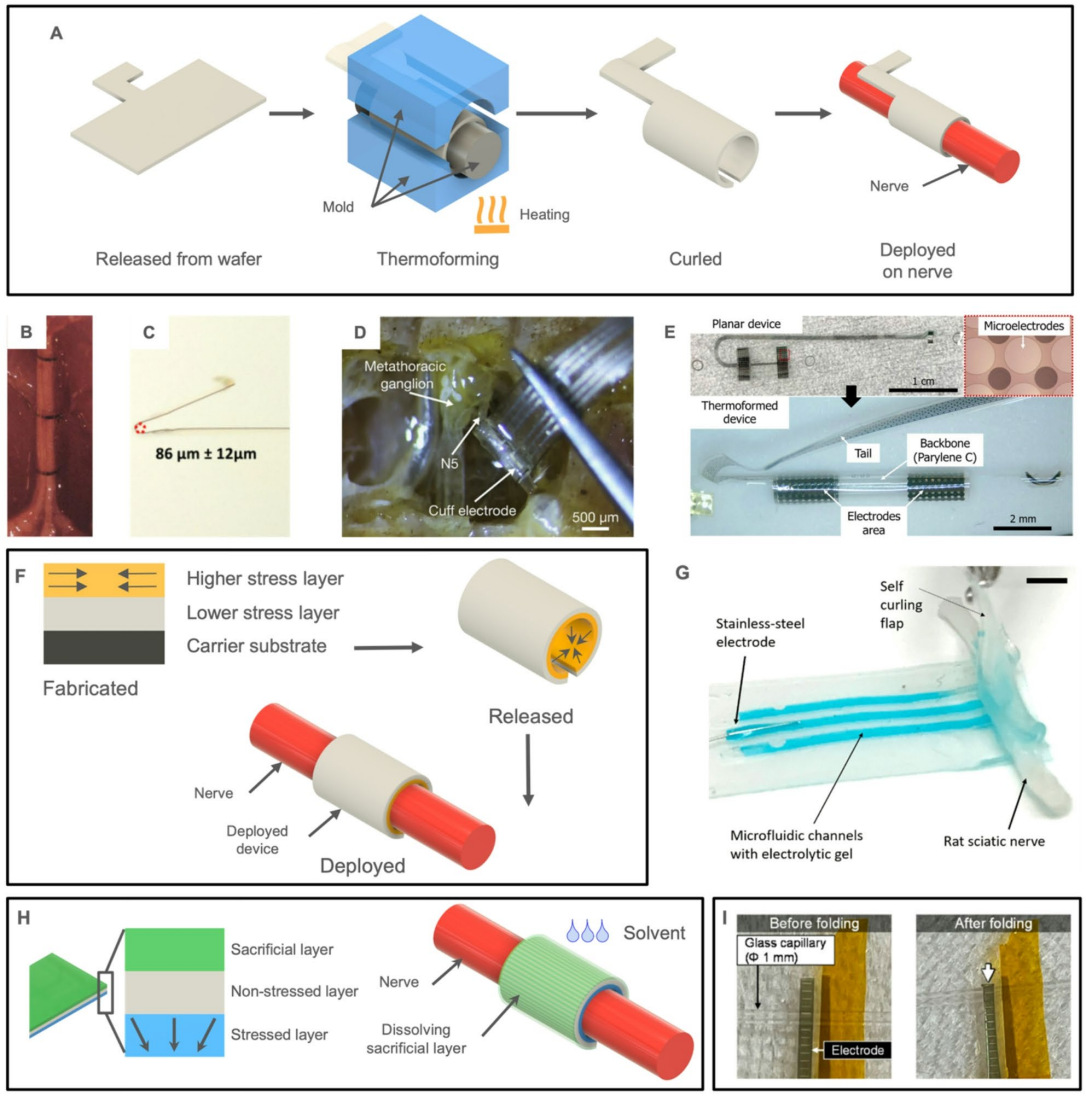
Examples of self-closing cuffs. (**A**) Illustration of thermoforming process to produce a self-closing cuff. From left to right, a planar device released from a carrier wafer, thermoforming using a mold and heat to achieve the final shape, release from the mold, and self-closing device deployed on nerve. (**B**) A thermoformed tripolar polyimide cuff electrode implanted on the rat sciatic nerve. Adapted from (Rodriguez et al. 2000) with permission from Elsevier. Copyright 2000 Elsevier. (**C**) A thermoformed polyimide cuff electrode of 86 μm diameter to interface with the vagus nerve of rat. Adapted from (Sun et al. 2023) with permission from Elsevier. Copyright Elsevier 2023. (**D**) A multichannel thermoformed Parylene C cuff implanted on the locust N5 nerve. Adapted from (Zurita et al. 2024) under CC BY 4.0. (**E**) A Parylene C based self-closing cuff designed for clinical applications targeting nerves less than 1 mm in diameter. Reprinted with permission from (Rezard et al. 2025). Copyright 2025 IEEE. (**F**) Illustration of self-closing arising from response to residual stress differences in the device layers after release from a stiff carrier substrate. (**G**) A multilayer, self-closing silicone cuff electrode with hydrogel electrolyte traces and electrodes around a mock nerve (scale bar = 5 mm). Reprinted from (Thakur et al. 2021) under CC BY 4.0. (**H**) Illustration of on-demand self-closing structure. Left: multilayered structure composed of a sacrificial layer, low residual stress layer, and high residual stress layer. Right: self-closing on a nerve triggered by dissolution of the sacrificial layer to release residual stress. (**I**) A self-closing graphene-based thin-film electrode folding around a glass capillary as a mock nerve. Left: device in a planar configuration before folding. Right: device folded around the mock nerve after dissolving calcium alginate gel sacrificial layer with ethylenediaminetetraacetic acid. Reproduced from (Goto et al. 2025) under CC BY 4.0

Since a clip adds bulk to the device profile, self-closing cuff concepts that avoid the use of sutures and manual wrapping have been developed (Fig. 8). This is largely achieved by pre-forming thin film cuffs into the final curled configuration before applying to a nerve (Fig. 8a). A flat polyimide-metal-polyimide film was configured into a cuff format through the application of heat while in the rolled-up form and applied to rat sciatic nerve (~ 1.2 mm diameter, Fig. 8B) (Rodriguez et al. 2000). A similar approach was used to create a cuff for the vagus nerve of rats with diameters as low as 86 μm (Fig. 8 C) (Sun et al. 2023). Sometimes referred to as thermoforming, this technique can also be applied to thermoplastic Parylene C (B. J. Kim et al. 2014). A thermoformed Parylene cuff (150 μm diameter) was implanted on the N5 nerve of locusts over 2 weeks with no impairment in insect activity observed (Fig. 8D) (Zurita et al. 2024). Another self-closing cuff intended for clinical applications targeting nerves less than 1 mm in diameter was reported having an aggregate electrode structure and electrodeposited coatings; both recording and stimulation were reported in acute studies on rat sciatic nerve (Fig. 8E) (M. Li et al. 2025; Rezard et al. 2025).

Self-curling of a planar multi-layer thin film device can be achieved by controlling residual stress in different layers of successively deposited Parylene C (Fig. 8 F). Once released from the carrier substrate, the film curls in response to the differential stress to form a cuff (Kang et al. 2015; Thakur et al. 2021) (Fig. 8G). The cuff shape can also be formed on demand and the direction of curling controlled using timed chemical dissolution of a retaining sacrificial layer under an intentionally structured film (Goto et al. 2025) (Fig. 8H). A stress difference can be achieved by freezing stretched polyvinyl alcohol hydrogel before bonding to an unstretched layer. Once thawed, the device curls towards the stretched side, forming a cuff (Terutsuki et al. 2022) (Fig. 8I).

#### Actuating cuff electrodes

**Fig. 9:**
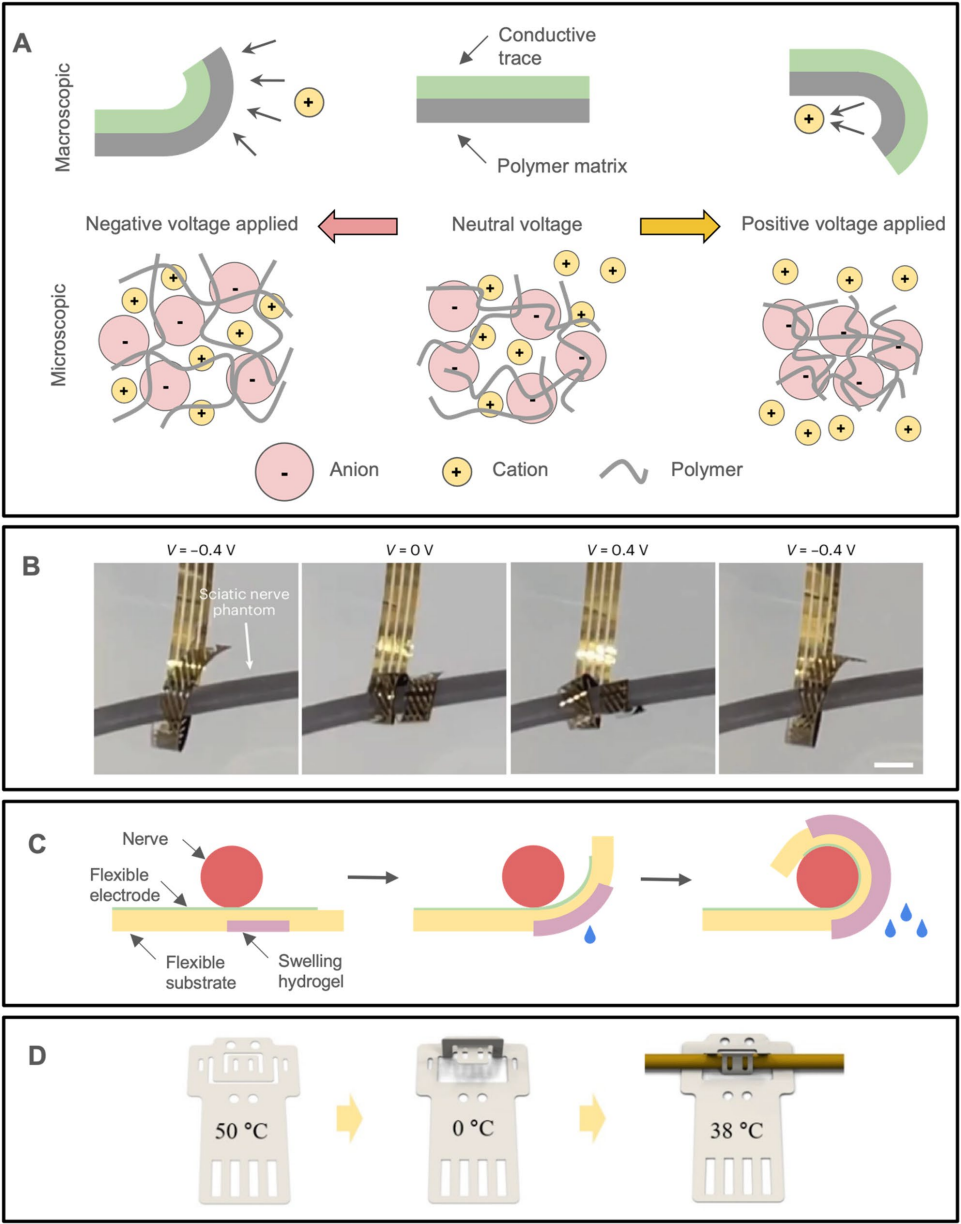
Examples of actuating mechanisms for nerve cuffs that do not require direct handling. (**A**) Illustration of possible states in electrochemically activated nerve cuff electrodes. The cuff bends when the polymer swells and shrinks, in response to an applied voltage that induces cations to enter or exit the polymer matrix. (**B**) An electrochemically actuated cuff electrode wrapping around a 1.4 mm diameter nerve phantom at different applied voltages. (Dong et al. 2024) under CC BY 4.0. (**C**) Illustration of the activation of a nerve cuff with a swelling hydrogel. The hydrogel swells in response to water to curl the cuff electrode around the nerve. (**D**) Illustration of a shape memory polymer nerve cuff that activates near body temperature. The device is flat at 50 ˚C, and 3-dimensional clipping features are introduced at 0 ˚C. At body temperature, which is closer to 50 ˚C, the device tries to return to the flat configuration and ends up curling around the nerve. Adapted from (Cho et al. 2021) under CC BY 4.0

Instead of relying on mechanical means for self-closing, a chemical or thermal actuation mechanism can be incorporated so that the cuff wraps itself around the target nerve without direct handling (Fig. 9). A volumetric change involving the movement of ions in and out of an electrochemically activated polymer (polypyrrole doped with dodecylebene sulfonate) has been used to reversibly curl and uncurl a cuff and record activity from the rat sciatic nerve (Fig. 9A-B) (Dong et al. 2024). A swelling hydrogel can be integrated into an initially flat cuff. Upon fluid contact, the hydrogel will swell, creating a mismatch in internal stresses to close the device around the nerve (Yu et al. 2023). This process is reversible since hydrogels expand when wet and contract when dry (Fig. 9 C). The thermally induced shape change of SMPs can be used to wrap an electrode array around a nerve at body temperature (Hiendlmeier et al. 2023; Zhang et al. 2019). First, a cuff electrode is designed to be completely closed at 50˚C and completely opened at 0˚C. After implanting, where the temperature is 37˚C, the device will transform to its closed state (Fig. 9D) (Cho et al. 2021).

#### Interfaces integrating microchannels

**Fig. 10:**
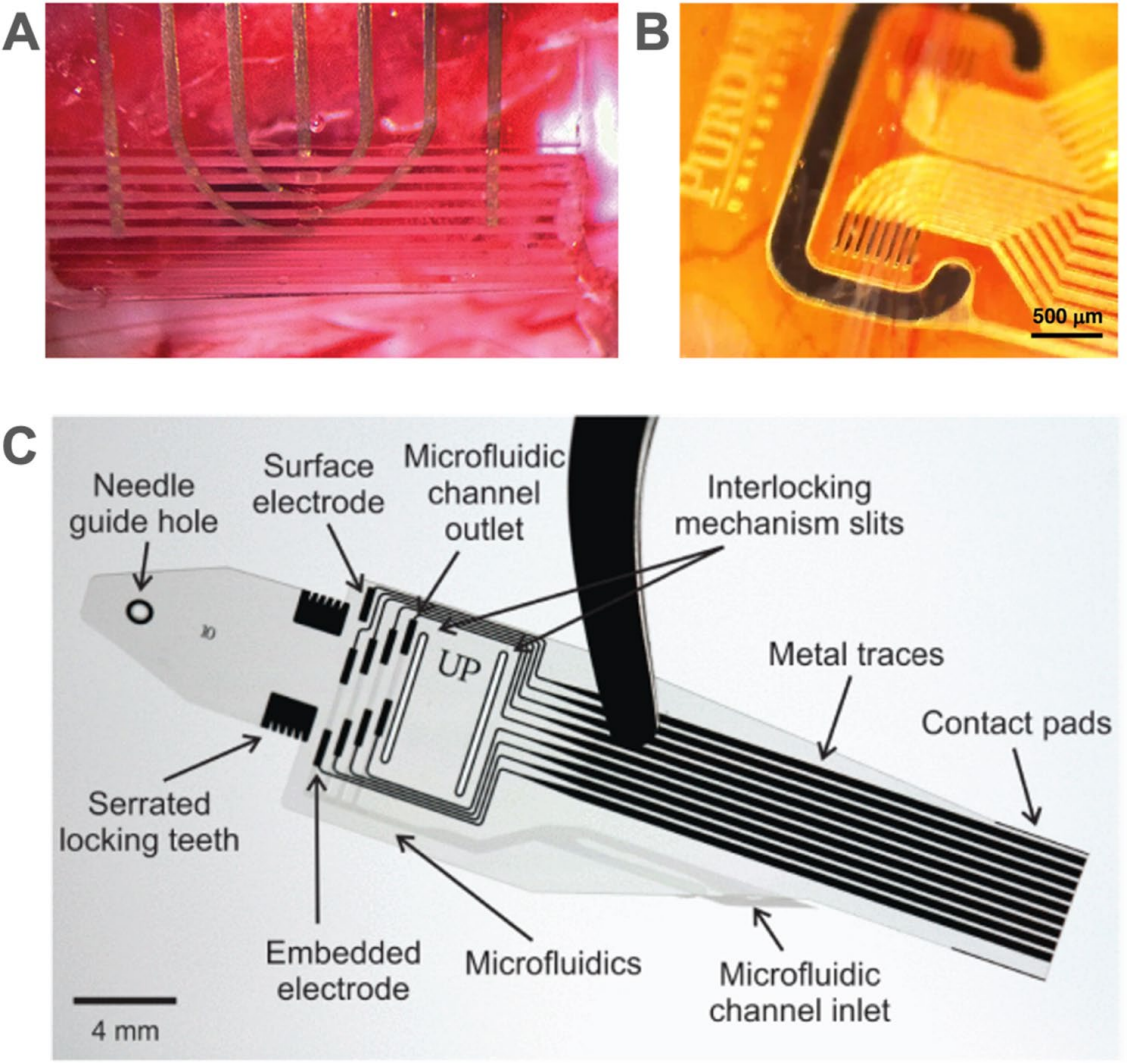
Examples of peripheral nerve interfaces in which the nerve is teased into distinct bundles that are then interfaced with electrodes. (**A**) L6 dorsal rootlets of rat teased and placed into 100 μm wide PDMS microchannels to detect bladder fullness. Adapted from (Chew et al. 2013) with permission from the American Association for the Advancement of Science. Copyright 2013 The American Association for the Advancement of Science. (**B**) An array of 40 μm wide polyimide microchannels containing teased branches of rat ventral gastric branch of the vagus nerve. Adapted from (Lim et al. 2024) under CC BY 4.0. (**C**) Fabricated lyse-and-attract cuff electrode (LACE) device. Reproduced from (Cobo et al. 2019) with permission from IEEE. Copyright 2019 IEEE.

Miniaturized book electrodes can contain tiny channels that interface with nerve segments surgically separated from a larger nerve. A molded PDMS microchannel device (each 100 μm wide channels) was developed to interface with the teased dorsal rootlets of rats and obtain information on bladder fullness (Fig. 10A) (Chew et al. 2013; Delivopoulos et al. 2012). A thin film microchannel device (11.5 μm thickness) having polyimide and SU-8 construction achieved 40 μm wide microchannels to interface with teased nerves from the ventral gastric branch of the vagus nerve (~ 100 μm diameter) of rats, demonstrating recording of activity from the unmyelinated C-fibers (Fig. 10B) (Lim et al. 2024).

Anti-inflammatory agents such as dexamethasone may be administered to mitigate the FBR to implants (De La Oliva et al. 2018) and can be eluted by modified electrodes or insulating substrates (Turrin et al. 2025). Controlled release of dexamethasone was demonstrated in functional nerve cuff electrodes using nanofibers and hydrogels (FitzGerald 2016; Park et al. 2015). Additionally, anti-inflammatory cyclosporin A was incorporated into electrodes with polyethylene glycol hydrogels for interfacing with the rat sciatic nerve (Heo et al. 2016). Anesthetics such as lidocaine can be administered via nerve interfaces to temporarily inhibit peripheral nerve function. For example, nerve blocking was achieved in rabbits and macaques using a silicone elastomer embedded with stimulating wires and an injection dome to induce anesthesia with lidocaine (Pohlmeyer et al. 2009).

Microfluidic channels were integrated into a Parylene C cuff electrode to enable delivery of lysing agents to selectively remove the epineurium and then induce axonal growth in fascicles through the subsequent release of growth factors with the goal of improving selectivity (Fig. 10C). Microfluidic release and effectiveness of the lysing agent was demonstrated on rat sciatic nerve (Cobo et al. 2019; Elyahoodayan et al. 2020).

### Intraneural and intrafascicular small nerve interfaces

Intraneural interfaces for small nerves present an additional surgical challenge as electrodes must be advanced past the epineurium to gain access to fascicles. Multiple device formats and implantation strategies have been explored to achieve intraneural access while managing the degree of invasiveness and minimizing injury to the nerve and surrounding tissues. The primary strategies employed are inserting an interface perpendicular or in parallel to nerve fibers.

#### Wires, probes, and yarns

**Fig. 11:**
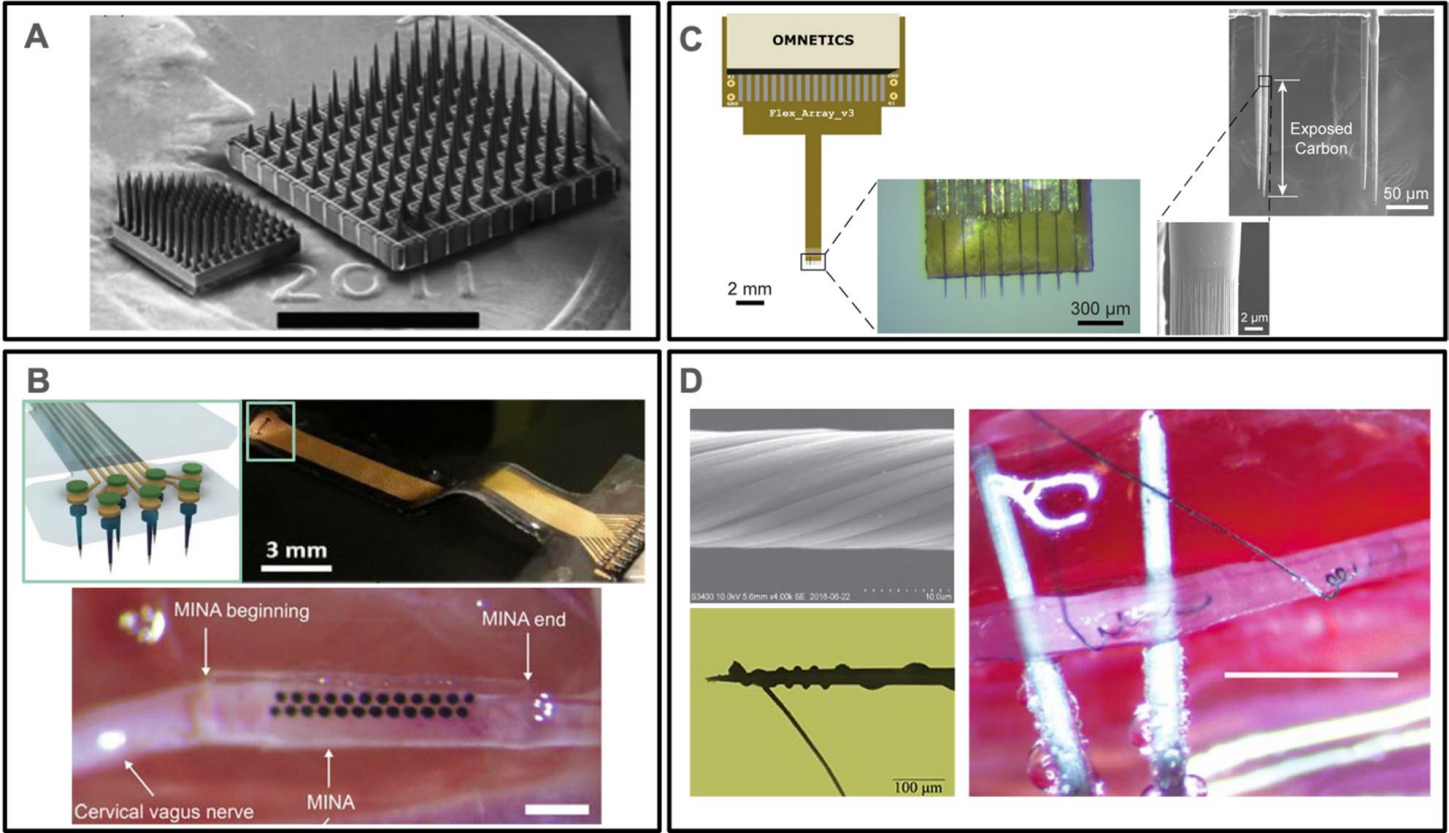
Examples of intraneural interfaces based on probes, wires, and yarns. (**A**) SEM of conventional Utah slanted electrode array (USEA) (right) along with high-density USEA (left) on a US penny for scale reference (scale bar = 3 mm). Adapted from (Wark et al. 2013) under CC BY 3.0. (**B**) Top row shows an illustration of the tip of a flexible microneedle nerve array (MINA) on the left and photograph of the overall device with the tip region boxed on the right. The bottom row shows the MINA attached onto a vagus nerve (scale bar = 500 μm). Adapted from (Yan et al. 2022) with permission from John Wiley and Sons. Copyright 2022 John Wiley & Sons. (**C**) Illustration (left), photograph (left inset), and SEMs (right and right inset) showing different views of a carbon fiber microelectrode array with 16 carbon-fiber electrodes. Adapted from (Jiman et al. 2020) under CC BY 4.0. (**D**) Top left shows an SEM image of CNT yarn. Adapted from (Zhu et al. 2019) under CC BY 3.0. Bottom left shows the CNT yarn wrapped around a tungsten microwire to facilitate easy insertion into the nerve. Adapted from (Kotamraju et al. 2023) under CC BY 4.0. On the right is an image of two CNT yarns implanted in the rat vagus nerve (scale bar = 2 mm). Adapted from (McCallum et al. 2017) under CC BY 4.0.

Single site microwires, single site silicon probes, and multisite silicon probes have all been deployed as intraneural interfaces by inserting across the epineurium perpendicular to nerve fibers. An array of 16 Parylene-insulated tapered iridium microwires having two different lengths and exposed AIROF tips were integrated with a silicone nerve cuff. The sharp tips could be directly inserted with gentle manual pressure into rat sciatic nerve (Troyk et al. 2015). Similarly, needle-like silicon probes can also be directly inserted into peripheral nerves. The Utah array (Fig. 11A), consisting of a high-density array of tapered silicon needles (25 electrodes/mm^2^) each with SIROF coated tips and having a gradual gradient of lengths (slant array), was pneumatically inserted at high velocity into rat sciatic nerve (1.2 mm diameter) and cat pudendal nerve (1 mm diameter) without evidence of nerve crush injury (Wark et al. 2013). However, in a follow up chronic study, a decellularized organic nerve wrap was used to keep the array in place on rat sciatic nerve and the initial nerve dysfunction attributed to insertion trauma was resolved within 2–3 weeks (Wark et al. 2014, p. 201). The feasibility of stimulation and recording using this device for patients with limb amputations is being evaluated clinically (“Clinical Trial: Can an Array of Micro-Electrodes Implanted in a Human Nerve Record Neural Signals and Provide Feedback?,” 2022; George et al. 2019).

An alternate method was used to produce the microneedle probe array, embedding tapered microneedles (~ 13–15 μm diameter at the center) in a soft silicone (Fig. 11B), rather than rigid silicon back plane, thus realizing a flexible probe array to access smaller nerves such as the rat peroneal nerve (George et al. 2019; Yan et al. 2022). To interface with sub-millimeter autonomic nerves, the use of linear carbon fiber arrays attached to a printed circuit board backbone was explored (Fig. 11 C). The small diameter fibers (8–9 μm in width and 150–250 μm in length) were insulated with Parylene C, sharpened to conical tips using a blowtorch, and the tips were coated with PEDOT: sodium p-toluenesulfonate (PEDOT:pTS). Tips could penetrate rat cervical vagus nerve (300–500 μm diameter) and record nerve activity (Jiman et al. 2020).

Microwires can also serve as a shuttle to insert CNT yarn electrodes, made from vertically aligned multiwalled CNT yarn (10–20 μm diameter) joined to 35NLT-DFT lead wire with conductive epoxy and encapsulated with Parylene C (Fig. 11D) *(*Kotamraju et al. 2023; McCallum et al. 2017; Zhu et al. 2019). The yarn electrode was wrapped around a tungsten microwire used as an insertion shuttle and advanced into the nerve directly or into individual fascicles exposed by dissecting the epineurium. The tungsten microwire was then withdrawn, leaving the yarn electrode within the nerve, and fibrin glue was used to secure the wire. Yarn electrodes were demonstrated in glossopharyngeal, vagus, and sciatic nerves of rats.

In addition to perpendicular insertion of wires and probes, wires have also been threaded along fascicles, then introduced into and pulled out through the epineurium with a needle. These longitudinal intrafascicular electrodes (LIFE) typically use Pt or PtIr wire or metallized Kevlar insulated with silicone; portions of the insulation are removed to create electrode contacts within the nerve (Malagodi et al. 1989).

#### Thin film devices

Wires and probes having a single conductor provide limited access to neural tissue and can be modified using microfabrication to allow use of the entire length to support multiple electrode sites. This approach was demonstrated in flexible thin film LIFE devices, enabling multiple electrode sites along the length and compliant polymer support structure (Lago et al. 2007; Lawrence et al. 2004). A thin film LIFE having electrodes on both planar surfaces was produced by mirroring the design and folding in half. The folded region provided a loop to guide the thread-like structure into and along the nerve. However, this surgical implantation process can be challenging if multiple devices must be placed (Thota et al. 2015).

To gain access to a larger number of fascicles in a single implantation surgery, thin film threads having multiple electrodes inserted perpendicularly relative to the nerve have been developed. These transverse intrafascicular multichannel electrodes (TIME) were fabricated in polyimide, folded to support electrodes on both sides of the thread (280 μm wide), and achieved muscle activation via stimulation of rat sciatic nerve (Boretius et al., 2010). Over a period of 2 months, TIME electrodes were observed to stay in place, were associated with mild encapsulation, and did not compromise motor or sensory functions in the implanted limb (Badia et al. 2011). In clinical trials, TIME was placed in the median and ulnar nerves of hand amputees to provide sensory feedback for controlling a hand prosthesis (Raspopovic et al. 2014) and transradial amputees for investigating the potential of multi-channel simultaneous stimulation to restore sensory feedback (Strauss et al. 2019).

**Fig. 12:**
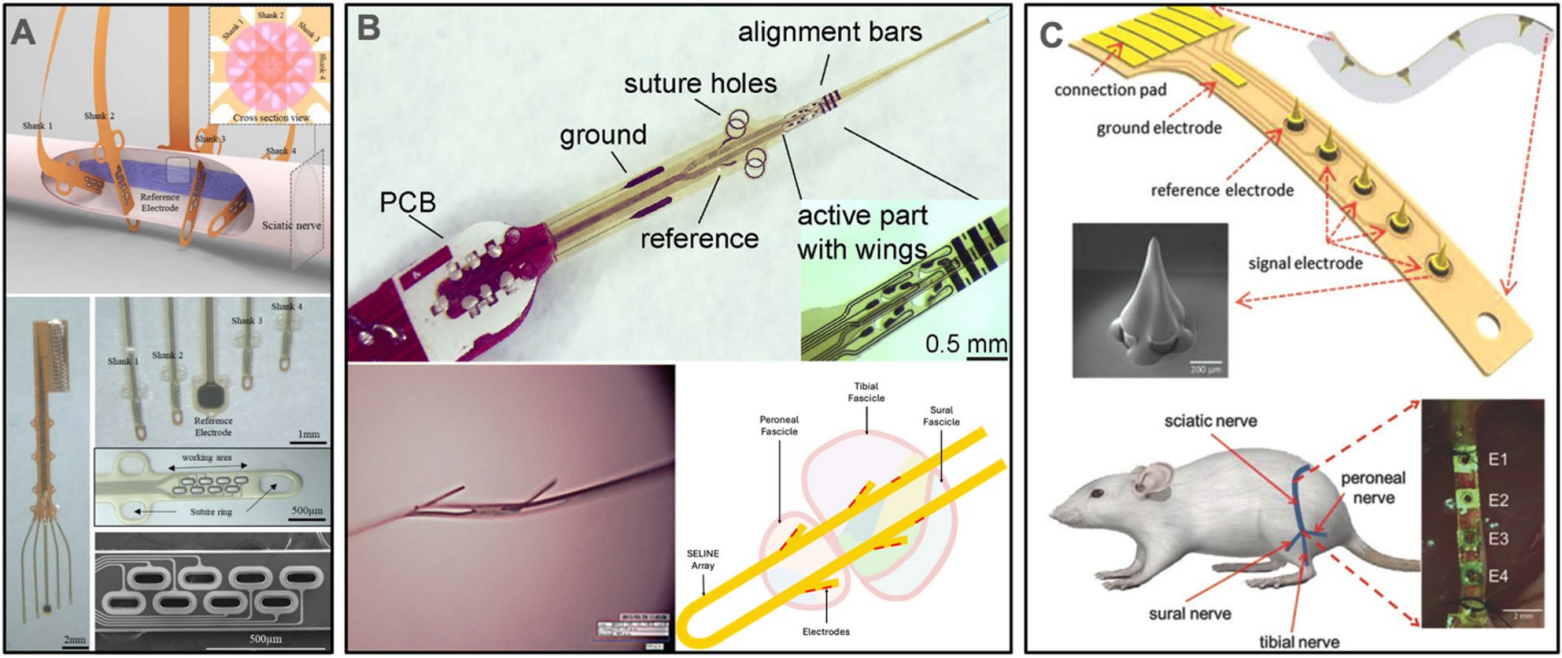
Examples of thin film intraneural interfaces. (**A**) (top) Illustration of fork-shaped neural interface having eight electrodes per shank with inset showing the distribution of electrodes after implanted spirally into a sciatic nerve. (bottom) Photographs of the fork-shaped neural interface (left), reference electrode and four shanks (top), with each having eight ring electrodes (middle), and SEM image of the ring electrodes (bottom). Adapted from (Choi et al. 2024) et al. with permission from IOP Publishing. Copyright 2024 IOP Publishing. (**B**) (top) A self-opening intraneural peripheral nerve interface and inset showing a detail of the anchoring wings. Adapted from (Wurth et al. 2017) with permission from Elsevier. Copyright 2017 Elsevier. (bottom left) Electrode wings deployed out-of-plane. Adapted from (Cutrone et al. 2015) with permission from IOP Publishing. Copyright 2015 IOP Publishing. (bottom right) A schematic of the SELINE array implanted in the rat sciatic nerve. (**C**) (top) Illustration of a thin film neural interface with spiked electrodes and insets on the left bottom showing SEM of the spike electrode and top right illustrating application of the device along a nerve. (bottom) Drawing of a rat showing position of the interface on the left sciatic nerve and inset showing device implanted on sciatic nerve. Adapted from (Wang et al. 2018a) with permission from John Wiley and Sons. Copyright 2018 John Wiley & Sons.

The TIME concept was extended to a fork-shaped polyimide device with four intrafascicular threads, each inserted independently and containing eight ring electrodes, perforated centrally to promote regeneration across the thread in rat sciatic nerve (Fig. 12A) (Choi et al. 2024a, b). Each shank was inserted at 45° with respect to the previous one along the length, guided by a 3D printed fixture, resulting in a spiral pattern of inserted threads to distribute electrodes more uniformly across the nerve.

To prevent the potential displacement of TIME-like devices which have no anchoring features, a self-opening intraneural peripheral interface (SELINE) was developed which included out-of-plane “wings,” or barb-like structures, that were activated by retracting slightly after inserting the device (Fig. 12B) (Cutrone et al. 2015). Although earlier studies confirmed that SELINE achieved greater stability when compared to TIME in rat sciatic nerve, a longer 6 month study revealed an initial increase in impedance and stimulation threshold that then stabilized, potentially attributed to the observed encapsulation, acute structural damage, and partial demyelination of axons (Wurth et al. 2017).

Repositioning of electrodes in the brain using microdrives has been successfully used for several decades, stemming from early reports of manual microdrives (Morrow, 1980) to widely available commercial systems. A similar concept was applied to improve signal quality and overcome the effects of fibrotic encapsulation using TiNi shape memory alloy actuators that operate based on joule heating integrated into the thin film LIFE devices (Bossi et al. 2009). A maximum displacement of 25 μm could be applied to reposition electrodes and was limited by maintaining operation within safe temperature limits (Bossi et al. 2006).

Microfabricated electrode sites are typically recessed since the conductor is typically sandwiched between layers of insulation. Using additional fabrication steps, it is possible to add material to the electrode sites, causing them to protrude from the surface and therefore, be positioned closer to fascicles. A stiff material for electrode sites such as vertically aligned 3D carbon nanotubes may be favorable as it can fill in the gap between the nerve and the cuff and penetrate the epineurium. Testing on the rat sciatic nerve showed good recording and stimulation capabilities as the device was conformally situated with the aid of the flexible Parylene C substrate (Tian et al. 2018). A linear array of conical microneedles was fabricated on top of planar electrode sites contained within Parylene wrapped polyimide thin films (Fig. 12 C). The microneedles were built up using SU-8, a photosensitive epoxy, using a double-draw lithography process, and then the four cones coated in thin film gold through a shadow mask. Spike electrodes were implanted one at a time by nicking the epineurium before pressing the spike into the rat sciatic nerve (Wang et al. 2018a, b).

### Regenerative small nerve interfaces

Since the 1880 s, peripheral nerves have been reconstructed via nerve allografting, in which two transected nerve stumps are joined together with a transplanted section (Schmidt 1993). Regenerative interfaces leverage the ability of fibers from transected nerves to extend and reconnect in the presence of growth factors and directly incorporate electrodes within the nerve (Spearman et al. 2018; Zellmer and Moran 2021). Since regenerative PNIs require nerve transection and regrowth occurs over 2–3 weeks with complete stabilization requiring ~ 6 weeks, applications are more limited (Polasek et al. 2009; Yang et al. 2008). Regenerative PNIs can be categorized into sieve, multichannel, and scaffold form factors.

#### Sieve

**Fig. 13:**
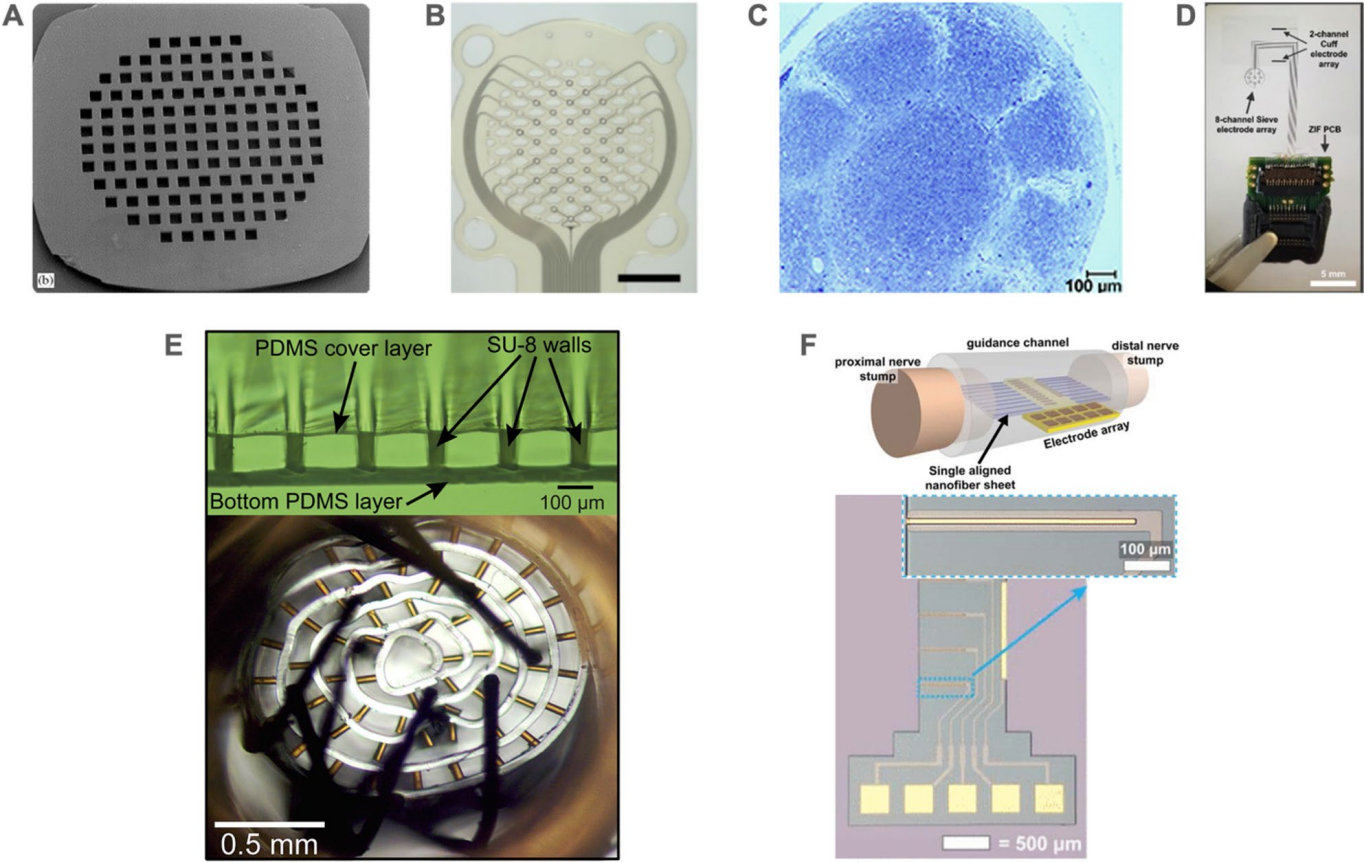
Examples of regenerative nerve interfaces. (**A**) An SEM of a silicon micromachined silicon sieve with 90 μm diameter holes. Adapted from (Wallman et al. 2001) with permission from Elsevier. Copyright 2002 Elsevier. (**B**) A micromachined polyimide sieve electrode with 30% porosity. Adapted from (Choi et al. 2024) under CC BY 4.0. (**C**) Toluidine blue stained axons regenerated in a macro sieve electrode (MacEwan et al. 2016) under CC BY 4.0. (**D**) An interface having both an epineural cuff and sieve electrodes. Adapted from (Kim et al. 2020) with permission from Elsevier. Copyright 2020 Elsevier. (**E**) (top) Micrograph of a microfabricated microchannel array formed by SU-8 walls and horizontal PDMS layers. (bottom) Micrograph of rolled microchannel scaffold array ready for implantation with integrated wire electrodes for electrophysiology. Adapted from (Srinivasan et al. 2015) with permission from Elsevier. Copyright Elsevier 2014. (**F**) (top) Illustration of a regenerative scaffold electrode array integrated with aligned nanofibers to guide nerve growth along the length of the electrodes. (bottom) Micrograph of a 4-channel recording electrode array on a Si wafer. Inset is a magnified view of one recording electrode. Adapted from (Clements et al. 2013) with permission form IEEE. Copyright IEEE 2013

The sieve electrode involves the integration of a porous structure supporting electrode sites into a transected nerve such that the nerve regenerates through the pores in the sieve. Earlier reports investigated the use of ceramic, Teflon, epoxy, and silicon for the sieve material, with silicon emerging as the preferred material for a time, since microfabrication techniques allowed precise control over pore placement and geometry (Fig. 13A) (Kawada et al. 2004; Kovacs et al. 1992; Navarro et al. 1996; Zellmer and Moran 2021). Then, polymers such as polyimide were adopted to reduce the mechanical stiffness while still allowing for pore control via microfabrication (Fig. 13B) (Choi et al. 2024b; Stieglitz et al. 1996, 1997). The functional outcomes of sieve electrodes are heavily dependent on their geometric design including hole sizes, hole spacing, transparency, and number of electrodes. Smaller hole size can limit the number of axons and thereby improve selectivity (Ceballos et al. 2002; Choi et al. 2024b, p. 20; Zellmer and Moran 2021).

An alternate approach used large openings, creating a macrosieve with fewer electrodes for rat sciatic nerve and demonstrating robust regeneration (Fig. 13C) (MacEwan et al. 2016). A hybrid approach merging the cuff and sieve strategies in a single microfabricated thin film device was reported in acute experiments on rat sciatic nerve; after transected nerve stumps were positioned across the sieve, the device was folded at 90˚ at the one edge of the sieve to position the wrap flaps to form the extraneural cuff (Fig. 13D) (Kim et al. 2020).

#### Microchannel

To guide axon regeneration and provide signal confinement, electrodes can be integrated into microchannel bundles which take the place of a planar sieve (Coker et al. 2019; FitzGerald et al. 2008). Development was driven by the need for improved nerve interfaces for use with mechatronic prosthetic arms (Larson and Meng 2019; Zellmer and Moran 2021). Guidance for microchannel length generally follows ~ 1 cm for rat sciatic nerves and ~ 3 cm for small diameter nerves in humans (Schmidt & Leach, 2003). The percentage of open area defined by the channel inner diameters also plays a role in regeneration outcomes, with larger open area percentages producing more favorable results (Zellmer and Moran 2021).

Earlier devices relied on stiffer epoxy channels then shifted to softer materials such as Parylene C, polyimide, and silicone. Silicone channels can be produced by molding around a template consisting of a bundle of microwires; individual electrodes can then be inserted into the microchannels (Choi et al. 2014; Kim et al. 2015). This concept was demonstrated using a 5 mm long silicone block perforated with microchannels of two different diameters (75 and 200 μm) in rat sciatic nerve, although microchannel placement could not be precisely controlled or distributed (Choi et al. 2014). Improved control of microchannel distribution was achieved by rolling a planar microchannel array into a cylindrical structure (Lewitus et al., 2011). Parylene C microchannels (100 ✕ 40 ✕ 1500 µm^3^; W ✕ T ✕ L) were fabricated using a surface micromachining approach in which patterned photoresist served as the template for forming enclosed microchannels by sandwiching them in two layers of conformally deposited Parylene (Suzuki et al. 2006). Polyimide microchannels were microfabricated by creating trenches (100 μm wide) with embedded microfabricated electrodes. When the film was tightly rolled, a network of enclosed channels was formed (FitzGerald et al. 2012; Lacour et al. 2008). A similar concept was demonstrated with rat sciatic nerve using PDMS films with integrated electrodes and vertical SU-8 walls, producing rolled devices (3 mm long) with varying channel cross section dimensions (Srinivasan et al. 2015). Instead of rolling, it is also possible to create microchannel arrays by stacking sheets containing PDMS molded microchannels (Musick et al. 2015). Although planar microfabrication processes generally restrict channels to rectangular cross sections, one report suggests that shape may play an important role as the rectangular cross section was associated with more fibrous tissue (Lacour et al. 2009). It is important to note that increasing the number of electrodes in such formats can be challenging. One solution is to use thinned application specific integrated circuits containing the electrodes and circuitry for stimulation and recording along with microchannels to reduce the number of external wires required from the interface (Habibollahi et al. 2021, 2025).

#### Scaffold

Regenerating interfaces incorporating minimal “scaffold” material between nerve stumps have been developed to promote enhanced tissue integration. A device consisting of electrospun nanofibers adjacent to a planar electrode array and a semipermeable channel provided topological and spatial guidance cues for nerve growth (Fig. 13E) (Clements et al. 2013). In addition, temporary degradable scaffolding materials have been used. A set of polyimide electrode arrays resembling threads were embedded in a biodegradable hydrogel scaffold in the tissue-engineered electronic neural interface (TEENI) designed for upper limb amputees. The hydrogel was further wrapped in a sheet of small intestinal submucosa (Kuliasha et al. 2018; Nunamaker et al. 2017; Spearman et al. 2020) and later iterated on, adding microchannels created using magnetic alginate microparticles to guide nerve growth (magnetically aligned regenerative tissue-engineered electronic nerve interface, or MARTEENI) (Atkinson et al. 2022). Subsequent studies abandoned the hydrogel which degraded inconsistently but employed allografts to aid nerve regeneration (Smadi et al. 2024, 2025).

### Integrated wireless small nerve interfaces

**Fig. 14:**
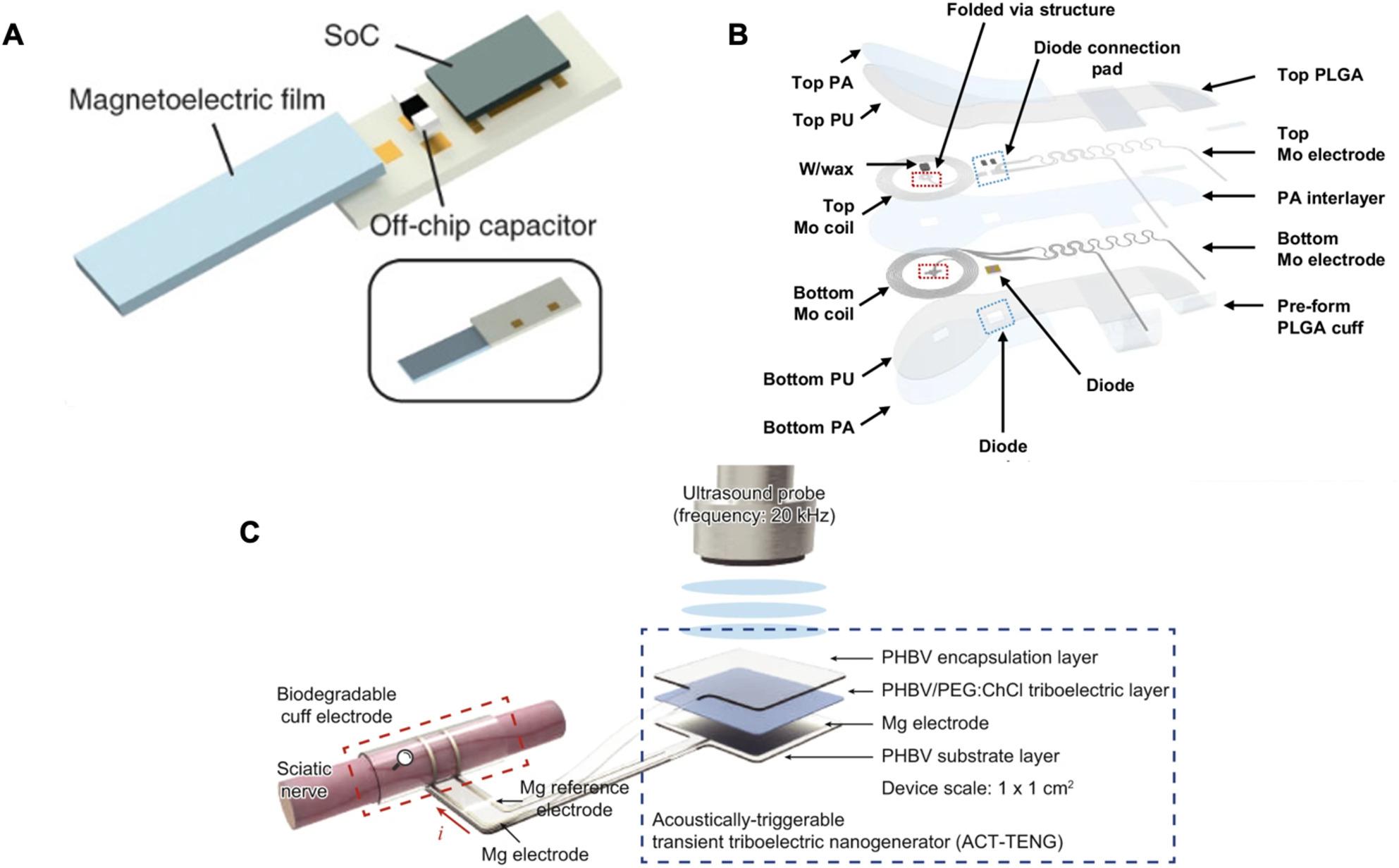
Examples of wireless peripheral nerve interfaces: (**A**) Illustration of the magnetoelectric powered bio-implant (ME-BIT) device consisting of a magneto-electric film, off-chip capacitor for energy storage, and system on chip (SoC) Reproduced from (Dorrian et al. 2024) under CC BY NC SA. (**B**) An exploded view illustration of an inductively coupled bioresorbable wireless stimulator for peripheral nerve regeneration. Reproduced from (Ahn et al. 2025) under CC BY NC ND. (**C**) Illustration of a biodegradable, wireless neurostimulator with an acoustically triggerable transient triboelectric nanogenerator shown in exploded view. Reproduced from (D.-M. Lee et al. 2023) under CC BY NC.

While many interfaces involve wires or leads to connect to distant or external electronics, advances in miniaturization and packaging have enabled millimeter scale fully integrated systems that include implanted electronics (Fig. 14A) (Dorrian et al. 2024). These integrated systems still require power to operate, and different strategies have been proposed. The StimDust (1.7 mm^3^ volume) integrated a piezoelectric transducer on a cuff electrode to stimulate the rat sciatic nerve with input from an external transceiver via an ultrasonic link (Piech et al. 2020). Piezoelectric materials can also transduce mechanical perturbation into a voltage output via an external shockwave system to promote the regeneration of axons (Tai et al. 2023). The ReStore wireless cuff employed standardized protocols such as near field communication and was tested chronically in canine cervical vagus nerve (Sivaji et al. 2019). A fully wireless floating microelectrode array stimulator was created by integrating microwire electrodes with an application specific integrated circuit, microcoil, and silicone cuff. Wirelessly controlled stimulation of the rat sciatic nerve resulted in observable muscle responses (Frederick et al. 2021). Different wireless communication techniques, including magnetic, transcutaneous ultrasound-driven triboelectricity inductive coupling, have been reported in bioresorbable peripheral nerve interfaces intended for temporary use as they are designed to be metabolized by the body (Fig. 14B**-**C) (Ahn et al. 2025; Koo et al. 2018; D.-M. Lee et al. 2023).

## Ongoing challenges in developing small peripheral nerve interfaces

The desire to develop interfaces that target smaller nerve branches is driven by the need to improve selectivity and avoid off-target effects. Current interfaces in FDA-approved clinical devices are too large in size, making them impractical for specific targeting of nerves smaller than 1 mm in diameter. In our perspective, although many experimental devices and different approaches to interfacing have been demonstrated in small animal models, with some making it to larger animal models and early human studies (Čvančara et al. 2020), translation of such devices into clinical practice will require addressing many challenges (Koutsouras et al. 2024). We identify several surgical and engineering challenges impeding the widespread availability and use of PNIs targeting smaller nerve branches.

### Surgical complexities of targeting smaller nerves

There is substantial intersubject variability which makes locating target peripheral nerves a challenge. Therefore, nerves must be localized preoperatively and intraoperatively for each subject. This is traditionally accomplished using magnetic resonance neurography (MRN) and ultrasound (US). MRN suppresses signals from fat and surrounding tissue while emphasizing those produced by nerves. Image quality can be impacted by movement or poor positioning of the patient (Chhabra et al. 2018). US utilizes the reflection of high-frequency sound waves from tissues and is limited to imaging superficial nerves. Thus, the two techniques are frequently used together. To optimize electrode placement, fluoroscopy, which enables real-time imaging, was used to place an electrode on the sacral nerve (Oerlemans and Van Kerrebroeck 2008). However, the risks of radiation exposure to both subjects and operators need to be considered (Müller et al. 2021).

Once the nerve is identified, the goal is to achieve precise surgical placement while minimizing any damage to surrounding tissues. Extraneural wires or cylindrical leads simply require insertion nearby the target nerve. However, cuff style interfaces require greater access, typically involving dissection from adjacent tissues in order to wrap the cuff around the nerve. Some self-closing cuff designs must be held open to accept a nerve and may require a specifically designed tool (Elyahoodayan et al. 2024). Intraneural, intrafascicular, and regenerative nerve interfaces, on the other hand, require access to the interior of the nerve and therefore place a greater burden on surgical dexterity and accuracy. As the dimensions of the target nerve decrease, the degree of surgical difficulty will tend to increase.

Advances in operative laparoscopy have led to the widespread use of minimally invasive surgery (Alkatout et al. 2021). Recently, laparoscopic techniques were used to deploy extraneural electrodes in minipig models and leads adjacent to nerves in humans (Løve et al. 2023; Rabischong et al. 2011). Peripheral nerve interfaces intended for laparoscopy delivery must be compatible with the gripper end effectors of laparoscopic surgical instruments, however thin film-based interfaces can be fragile, necessitating, in some cases, the addition of handling tabs. The design process should carefully consider the surgical placement method, integrating specific features to facilitate proper device implantation, and even the parallel design of custom insertion tools. Surgical robots have already facilitated the placement of cochlear leads in humans, where safety margins are sub-millimeter due to the complexity of adjacent structures (Topsakal et al. 2022), and could potentially assist in placing delicate peripheral nerve interfaces.

Even if implantation is successful, natural movements may induce device migration which may be further compounded by device features (Carnicer-Lombarte et al. 2021; Paggi et al. 2024). Clinical and experimental devices have explored the use of tines, wings, and other anchoring structures. Adding strain relief loops may help relieve tension on leads and prevent transmission of forces that may dislodge a device. However, there exist tradeoffs between rigidly constraining a device relative to a nerve and allowing for some natural micromotion of the tissue to occur. Although not well documented in the published literature, the design process should also consider strategies for future removal and/or replacement of the implanted device without causing nerve damage (Howe et al. 2024).

### Engineering challenges associated with targeting smaller nerves

The number of axons in peripheral nerves (e.g., ~ 27,000 for rat sciatic nerve) far exceeds the number of electrodes per unit area (e.g., 56 for extraneural is considered high) currently achievable using available fabrication methods (Koh et al. 2019; Schmalbruch 1986). Therefore, a large amount of neural information is unresolvable and fiber phenotype is often indirectly determined via differences in conduction velocity (Lim et al. 2024). Even if electrodes could be produced in higher numbers, scaling physical connections to each electrode quickly becomes untenable. One solution is to locally integrate application specific integrated circuits that include multiplexing to reduce the number of connections but at the cost of not being able to access all channels simultaneously.

In addition, the move towards smaller footprint devices via reducing the volume of conductive and insulating materials increases electrode impedance and risks compromising passivation, leading to higher noise levels and potential signal leakage. This increases crosstalk and parasitic capacitance between neighboring channels and limits the performance and efficacy of the device. Computationally guided designs can improve outcomes but are limited by available materials (Rustogi et al. 2021).

Innovations in new materials that meet the demanding requirements for small scale PNIs are regularly introduced, enabling new interfacing formats and strategies. Experimental materials will need to transition to commercial viability as well as meet long-term biocompatibility requirements through extensive in vitro and in vivo testing if designed to be exposed to tissues (Hassler et al. 2011; Mariello et al. 2022). Such evaluation can be hindered by the inability to recreate in vitro the complex in vivo FBR mechanisms (Mariello et al. 2022) although computational simulations may assist in device development (Ciotti et al. 2023; Elder and Yoo 2018).

Implanted PNIs should be resistant to failure, whether arising from exposure to the in vivo saline environment or chronic stress induced by motion of tissue experienced during natural movements (Thielen and Meng 2023). Multi-layer construction common to microfabricated devices, in which dissimilar materials are joined often without strong chemical bonding, can be plagued by delamination, arising, for example, when the mechanical stress at the interlayer interface exceeds the bonding forces (Ohring 2002). Interfacing to tiny nerves may push materials to their limits, inducing material fatigue due to the repeated mechanical motion of tissue or bending to accommodate very small diameters (Lee and Meng, 2015).

Long term use in which electrical stimulation is applied to thin film microfabricated electrode sites can also introduce additional failure modes. Oscillations arising from stimulation have been observed to cause damaging mechanical deformation on electrode sites (Schulte et al. 2024). Unintentional irreversible reduction and oxidation reactions can result in the electrolysis of water leading to toxic pH changes and dissolution of the electrode.

Any implanted material elicits FBR and will either be metabolized or encapsulated in fibrotic tissue. This is especially detrimental for the small nerve electrodes recording or stimulating small nerve fibers. In addition, fibrotic tissue may grow between the electrode contacts and adjacent target sites, leading to an increased impedance or a higher stimulation current required to obtain therapeutic effect, which may accelerate electrode failure. Some strategies, like drug coatings and electrode modification to reduce capsule formation (Rujitanaroj et al. 2013) have been employed to reduce the degree of protein adsorption on the electrodes. In addition, the inflammatory response can elicit the release of reactive oxygen compounds, initiating corrosive dissolution processes that degrade degrading electrode sites (Caldwell et al. 2020).

## Conclusion

Targeting peripheral nerves via electrode-based interfaces to deliver bioelectronic medical therapies has been of great interest since the 1960s but current clinical therapies with approved devices are limited to extraneural approaches and larger nerves. More distal peripheral nerves are intriguing as clinical targets, especially with our growing knowledge of peripheral nervous system physiology and anatomy and desire to avoid off-target effects associated with vagus nerve stimulation. Although available medical grade materials and manufacturing processes limited the achievable dimensions for traditional cuff-based electrodes, new advancements in materials, surface modifications, micro- and nanofabrication, closing and locking mechanisms, and wireless electronics offer new possibilities to realize interfaces for stimulation and recording of small nerves (Ø ≤ 1 mm). Many interconnected challenges remain before clinical grade devices for smaller nerves are ready and cross-disciplinary collaboration combining expertise and innovations in materials, fabrication, surgical technique, and tissue integration is required to devise holistic solutions to overcome them. Such collaborations are already in progress worldwide, with new interface concepts being explored and a few prospects already progressing in animal and early human studies. Continued progress in advancing small nerve interfaces is poised to open new therapeutic frontiers in bioelectronic medicines.

## Data Availability

No datasets were generated or analysed during the current study.

## Abbreviations

AIROF: Activated iridium oxide film
CIC: Charge injection capacity
CNT: Carbon nanotube
EIROF: Electrodeposited iridium oxide film
FBR: Foreign body response
IrOx: Iridium oxide
LCP: Liquid crystal polymer
LIFE: Longitudinal intrafascicular electrode
LM: Liquid metal
PDMS: Polydimethylsiloxane
PEDOT: Poly(3,4-ethylenedioxythiophene)
PNI: Peripheral nerve interface
PNS: Peripheral nervous system
PSS: Poly(styrene sulfonate)
PPy: Polypyrrole
RuOx: Ruthenium oxide
SELINE: Self-opening intraneural peripheral interface
SIROF: Sputtered iridium oxide film
SMP: Shape memory polymer
TEENI: Tissue-engineered electronic neural interface
TIME: Transverse intrafascicular multichannel electrodes
TiN: Titanium nitride
VNS: Vagus nerve stimulation

## Footnotes

1 Tensile strength of a material is determined by the maximum stress under stretching or pulling it can withstand before breaking.

2 Electrochemical impedance is the total opposition to alternating current. A lower value is more desirable.

3 Charge storage capacity is a measure of the maximum charge per unit area an electrode can store. A higher value is more desirable.

4 Charge injection capacity is a measure of the charge density per pulse that can be applied to an electrode before irreversible processes like electrolysis or electrode corrosion occur and without damaging tissue. A higher value is more desirable.

5 CSC is the theoretical performance value of an electrode material and can be more repeatably determined. However, CIC more accurately represents the actual charge that can be injected into tissue as neural stimulation, usually in the microsecond range, and relies more on the faster capacitive charge transfer, than the slower, surface adsorbed redox reactions (Cogan et al. 2016).
